# Biological and genomic characterization of *Escherichia* phage KBU_SV1 active against carbapenem-resistant *Escherichia coli*

**DOI:** 10.3389/fmicb.2026.1927801

**Published:** 2026-10-15

**Authors:** Sevil Öztaş, Abdulkerim Karaynir, Rumeysa Gulsu Ozkan, Bulent Bozdogan, Devrim Dündar

**Affiliations:** 1 Vocational School of Health Services, Karabük University, Karabük, Türkiye; 2 Recombinant DNA and Recombinant Protein Research and Application Center (REDPROM), Aydın Adnan Menderes University, Aydın, Türkiye; 3 VETAL Animal Health Products Inc., Adıyaman, Türkiye; 4 Department of Medical Microbiology, Faculty of Medicine, Aydın Adnan Menderes University, Aydın, Türkiye; 5 Department of Medical Microbiology, Faculty of Medicine, Kocaeli University, Kocaeli, Türkiye

**Keywords:** bacteriophage, biofilm, carbapenem resistance, *Dhillonvirus*, *Escherichia coli*, *Galleria mellonella*, phage therapy

## Abstract

Carbapenem-resistant *Escherichia coli* infections are increasingly difficult to treat, and bacteriophages have been highlighted as a promising tool to control such multidrug-resistant bacteria. Here, we isolated and characterized *Escherichia* phage KBU_SV1 using *E. coli* ATCC 25922 as the isolation and propagation host. Its morphology, host range, stability at various temperatures and pH, reduction growth of bacteria, effect on biofilm, *in vivo* efficacy, and genome were investigated. Transmission electron microscopy showed a polyhedral head and a long, non-contractile tail, corresponding to a *siphovirus* morphotype. The spot-test analysis of 106 bacterial strains showed spot clearance against 16 strains (host range index, 0.15), including carbapenem-resistant and carbapenem-susceptible *E. coli* isolates and, to a lesser extent, *Klebsiella pneumoniae*, while no such effect was observed in the other species tested. KBU_SV1 was stable between pH 4 and 12 and at 4–37 °C, but no infectivity was detected at pH 2 or 65 °C. Bacterial growth was suppressed at all multiplicities of infection (MOI) tested (100–0.01), followed by gradual regrowth. KBU_SV1 reduced established biofilms and inhibited biofilm formation in both *E. coli* ATCC 25922 and the carbapenem-resistant isolate Ec59. In the *Galleria mellonella* infection model, phage treatment significantly delayed larval mortality compared with the infection control, while no mortality occurred in larvae receiving phage alone. The genome of KBU_SV1 was found to be 45,190 bp with a G + C content of 53.56% and was complete with a 272-bp direct terminal repeat. The genome contains 71 putative open reading frames (ORFs) and no tRNA, while no virulence, antibiotic resistance (AMR) or lysogeny-associated genes were identified, and genome-based analyses predicted a virulent lifestyle. Comparative genomic analysis showed a high nucleotide identity (99.12%) with *Escherichia* phage EPIMAM03, and KBU_SV1 was assigned to the genus *Dhillonvirus* within the class *Caudoviricetes*. All these results showed that KBU_SV1 has favorable properties such as antibacterial potential against carbapenem-resistant *E. coli*, broad stability, antibiofilm and *in vivo* activity, and the absence of virulence and AMR genes. Based on all these features, this newly isolated phage is promising for the control of carbapenem-resistant *E. coli* infections, alone or in phage cocktails.

## Introduction

Antimicrobial resistance (AMR) has emerged as a “silent pandemic,” progressing steadily and posing one of the most serious global public health threats of the 21st century. Recent estimates indicate that approximately 1.27 million deaths worldwide in 2019 were directly attributable to infections caused by antimicrobial-resistant bacteria (Pal et al., 2024; Pirnay et al., 2024; Antimicrobial Resistance Collaborators, 2022). Surveillance data from the World Health Organization (WHO) Global Antimicrobial Resistance and Use Surveillance System (GLASS) highlight alarming resistance levels among clinically significant bacterial pathogens. These levels substantially compromise the effectiveness of commonly used antibiotics (Pal et al., 2024; World Health Organization Regional Office for Europe, 2022). Consequently, there is an urgent need for new antimicrobial strategies targeting priority pathogens identified by the WHO.

Despite substantial financial and regulatory incentives introduced to stimulate antibiotic discovery, the global development pipeline remains notably deficient in small-molecule agents with novel chemical scaffolds. A similar scarcity is observed among agents directed against innovative microbiological targets, across both preclinical and clinical (phase I–III) stages (Reza et al., 2025; Paterson, 2024). In response, non-traditional antibacterial approaches have attracted increasing interest, and approximately 41% of agents in the 2023 clinical pipeline belonged to this category. These include monoclonal antibodies, antivirulence compounds, bacteriophages and phage-derived enzymes, immunomodulators, and microbiome-modulating therapeutics (Reza et al., 2025; Paterson, 2024).

The 2024 WHO Bacterial Priority Pathogens List (BPPL) groups 15 families of antibiotic-resistant bacteria into critical, high and medium priority categories. Carbapenem-resistant Enterobacterales and third-generation cephalosporin-resistant Enterobacterales are listed as two separate items within the critical-priority group (World Health Organization, 2024). *Escherichia coli*, a member of this order, is among the most common human bacterial pathogens and causes enteritis, septicemia, neonatal meningitis, and urinary tract infections (UTIs). Extraintestinal infections, particularly UTIs, represent the most common clinical manifestation associated with this organism (Kaper et al., 2004; Gebremedhin et al., 2025). The increasing prevalence of multidrug-resistant (MDR) *E. coli* strains has complicated therapeutic management, contributing to prolonged hospital stays and escalating healthcare costs (Ruekit et al., 2022; Ochoa et al., 2016).

One of the major factors contributing to bacterial persistence and treatment failure is the ability of bacteria to form biofilms. Biofilms are structured microbial communities embedded within an extracellular polymeric substance (EPS) matrix that adheres to both biotic and abiotic surfaces (de Miguel et al., 2020). This architecture substantially reduces bacterial susceptibility to antimicrobial agents and enhances tolerance to host immune defenses, thereby complicating infection control and therapeutic outcomes. Consequently, the growing prevalence of MDR biofilm-forming bacteria has severely limited the effectiveness of conventional antibiotics. Moreover, prolonged antibiotic therapy may lead to adverse effects, disruption of the host microbiota, and further selection of resistant strains (Chegini et al., 2021; Taati Moghadam et al., 2020). Given these limitations, alternative antimicrobial strategies, particularly bacteriophage therapy, have attracted renewed attention (Chegini et al., 2020; Taati Moghadam et al., 2020).

Although an increasing number of *E. coli* phages have been described, phage susceptibility is strain-dependent, and relatively few well-characterized phages are available against carbapenem-resistant clinical isolates. Expanding the repertoire of fully characterized phages active against this critical-priority pathogen, including their genomic features, physicochemical stability, antibiofilm activity and *in vivo* efficacy, therefore remains an important need.

Accordingly, this study was designed to comprehensively evaluate a newly isolated bacteriophage active against carbapenem-resistant *E. coli* through genomic characterization, stability profiling, antibiofilm activity assessment, and *in vivo* efficacy studies. By integrating these approaches, the present work seeks to contribute to the growing body of evidence supporting bacteriophages as potential therapeutic agents against MDR *E. coli* infections and to provide insights that may facilitate the development of phage-based interventions for clinically relevant pathogens.

## Materials and methods

### Bacterial strains and culture conditions

All clinical isolates included in this study were obtained from the Clinical Microbiology Laboratory of Kocaeli University Hospital (Kocaeli, Türkiye). The carbapenem-resistant *Escherichia coli* (CREC) and *Klebsiella pneumoniae* (CRKP) isolates had been characterized in our previous study, in which their carbapenem resistance determinants, β-lactamase genes and antibiotic susceptibility profiles were identified (Öztaş et al., 2024). The CREC isolates carried multiple β-lactamase genes, including blaNDM, blaCTX-M, blaTEM, blaSHV, blaOXA-48 and blaVIM, and the CRKP isolates carried blaOXA-48, blaCTX-M and blaNDM (Supplementary Table S1). Several of these isolates were also resistant to last-resort agents, including ceftazidime–avibactam and plazomicin. Bacteria were routinely cultured at 37 °C in Luria–Bertani (LB) medium containing 5 g/L yeast extract, 10 g/L tryptone and 10 g/L NaCl (Merck, Germany), solidified with 1.5% agar (Biokar Diagnostics) when required.

### Bacteriophage isolation and enrichment

Phages were isolated from sewage water collected in Karabük, Türkiye. Isolation and enrichment were performed as described previously (Karaynir et al., 2022), with minor modifications. Briefly, 10 mL of sewage sample was centrifuged at 5,000 × g for 10 min at 4 °C to remove debris and particulate matter. The supernatant was filtered through a 0.22-μm pore-size membrane filter (Sartorius Stedim Biotech, Germany). To screen the filtrate for bacteriophages, aliquots were spotted onto top agar containing *E. coli* ATCC 25922 or *E. coli* ATCC 13846 and incubated overnight at 37 °C. Lytic activity was observed against *E. coli* ATCC 25922, which was subsequently used as the indicator and propagation host for KBU_SV1 throughout phage isolation, enrichment, purification, and titration. For enrichment, 10 mL of the filtered sewage sample was mixed with an equal volume of LB broth inoculated with 100 μL of an overnight culture of *E. coli* ATCC 25922 and incubated overnight at 37 °C. The enriched culture was centrifuged at 5,000 × g for 10 min at 4 °C, and the supernatant was filtered through a 0.22-μm membrane filter.

### Purification of isolated bacteriophages

Phage purification was performed by repeated plaque isolation using the double-agar layer method, as described previously (Karaynir et al., 2022). Soft agar was prepared using LB-broth with 0.5% agar and 5 mM CaCl₂. Serial dilutions of the phage lysate were prepared in SM buffer (100 mM NaCl, 8 mM MgSO₄·7H₂O, 50 mM Tris–HCl, pH 7.5, and 0.01% [w/v] gelatin). For plaque formation, 100 μL of each phage dilution and 100 μL of an overnight culture of *E. coli* ATCC 25922 were mixed with 4 mL of soft agar and poured onto LB agar plates. After overnight incubation at 37 °C, well-isolated plaques were selected according to plaque morphology and transferred into fresh LB broth containing *E. coli* ATCC 25922. Following overnight incubation, the lysates were centrifuged at 5,000 × g for 10 min at 4 °C and filtered through a 0.22-μm sterile membrane filter. Plaque purification was repeated four consecutive times to obtain a clonal phage preparation. Phage titers were subsequently determined using the double-agar layer method (Yang et al., 2010) and expressed as plaque-forming units per milliliter (PFU/mL).

### Host range determination of KBU_SV1 phage

The host range of KBU_SV1 was determined against a panel of 106 bacterial strains, comprising 20 CREC and 20 carbapenem-susceptible *Escherichia coli* (CSEC) clinical isolates, 40 CRKP clinical isolates, 20 *Enterococcus faecalis* clinical isolates, and six reference strains (*Staphylococcus aureus* ATCC 29213, *Enterococcus faecalis* ATCC 29212, *Pseudomonas aeruginosa* ATCC 27853, *Acinetobacter baumannii* ATCC 19606, *Escherichia coli* ATCC 25922, and *Klebsiella pneumoniae* ATCC 700603) (Supplementary Table S1).

Lytic activity was screened using a spot assay. Briefly, 100 μL of an overnight culture of each strain was mixed with 4 mL of soft agar and poured onto LB agar plates. After solidification, 10 μL of KBU_SV1 lysate (10^10^ PFU/mL) was spotted onto the bacterial lawn. Plates were incubated overnight at 37 °C and examined for visible zones of lysis (Melo et al., 2016). Strains producing a visible zone of clearing at the spot site were recorded as spot-test positive. The spot-assay-based host range index (HRI) was calculated as the number of spot-test-positive strains divided by the total number of strains tested.

### Morphological analysis by transmission electron microscope

The morphology of KBU_SV1 was examined by transmission electron microscopy (TEM). A 10-μL aliquot of concentrated phage stock (approximately 1 × 10^10^ PFU/mL) was applied onto carbon-coated copper grids (Pelco Grids, Redding, CA, USA). After 1 min, the excess phage suspension was removed with filter paper and the grid was negatively stained with 2% uranyl acetate (Merck) (Shahin and Bouzari, 2018). Grids were examined using a transmission electron microscope (HT7800, Hitachi, Tokyo, Japan), and the head diameter and tail length of the phage particle were measured using the instrument software.

### Thermal and pH stability of KBU_SV1 phage

The pH and thermal stability of KBU_SV1 were evaluated as previously described (Karaynir et al., 2022), with minor modifications. For the pH stability assay, 100 μL of phage lysate (10^9^ PFU/mL) was mixed with 900 μL of SM buffer adjusted to pH 2, 4, 6, 7, 8, 10, or 12 with HCl or NaOH. The mixtures were incubated at +4 °C for 60 min, after which the surviving phages were quantified using the double-agar layer method. For the thermal stability assay, phage suspensions prepared in SM buffer at approximately 10^8^ PFU/mL were incubated at 4, 23, 37, or 65 °C for 60 min. Following incubation, the surviving phages at each temperature were quantified using the double-agar layer method.

### Bacterial growth reduction assay

The bacterial growth reduction assay was performed as described previously, with modifications (Jamal et al., 2015). Briefly, an overnight culture of *E. coli* ATCC 25922 was diluted 1:100 in fresh LB broth, corresponding to an initial density of approximately 10^7^ CFU/mL. The diluted culture was immediately treated with KBU_SV1 at multiplicities of infection (MOIs) of 100, 10, 1, 0.1 and 0.01, calculated relative to this bacterial density (MOI = phage titer [PFU/mL]/bacterial density [CFU/mL]). To achieve these MOIs against an initial bacterial density of 10^7^ CFU/mL, corresponding phage concentrations of 10^9^, 10^8^, 10^7^, 10^6^, and 10^5^ PFU/mL were used. An untreated culture without phage addition was included as the growth control. Cultures were incubated at 37 °C, and optical density at 595 nm (OD₅₉₅) was measured every 2 h for 24 h using a spectrophotometer (Labomed Inc., USA), with sterile LB broth used as the blank. The experiment was performed independently in triplicate.

### Biofilm formation assay for *Escherichia coli* ATCC 25922 and clinical isolate

Biofilm formation by *E. coli* ATCC 25922 and the carbapenem-resistant *E. coli* (CREC) clinical isolate Ec59 (Supplementary Table S1) was evaluated using the crystal violet (CV) microtitre plate assay as described previously (Ali et al., 2023; Stepanović et al., 2007). Bacterial colonies were inoculated into fresh LB broth and incubated overnight at 37 °C. Overnight cultures were diluted 1:100 in LB broth supplemented with 0.25% glucose, and 200 μL of each diluted culture was transferred to a U-bottom 96-well microplate and incubated overnight at 37 °C. Uninoculated LB broth supplemented with 0.25% glucose was used as the negative control, and all conditions were tested in triplicate. Following incubation, planktonic cells were removed and the wells were washed three times with 200 μL of pre-warmed sterile phosphate-buffered saline (PBS; pH 7.4). The adherent biofilms were dried and fixed at 60 °C for 15 min and stained with 125 μL of 1% CV for 15 min. Excess stain was removed by rinsing with distilled water, and the plates were dried at room temperature. Bound CV was solubilized with 125 μL of a solution containing 80% (v/v) ethanol and 20% (v/v) acetone for 5 min at room temperature, and absorbance was measured at 595 nm (OD₅₉₅) using a microplate reader (Multiskan Spectrum, Thermo Fisher Scientific, USA). The optical density cut-off (ODc) was defined as the mean OD₅₉₅ of the negative control plus three standard deviations. Isolates were classified as non-biofilm producers (ODs ≤ ODc), weak (ODc < ODs ≤ 2 × ODc), moderate (2 × ODc < ODs ≤ 4 × ODc) or strong biofilm producers (ODs > 4 × ODc), where ODs is the OD₅₉₅ of the sample.

#### Reduction of established biofilms and inhibition of biofilm formation by KBU_SV1

The activity of KBU_SV1 against established biofilms and its ability to inhibit biofilm formation were assessed as described previously with modifications (Ali et al., 2023; Karaynir et al., 2022). In both assays, KBU_SV1 was applied at multiplicities of infection (MOIs) of 100, 10, 1, 0.1 and 0.01, calculated relative to a bacterial density of 10^8^ CFU/mL.

For the treatment of established biofilms, bacterial suspensions (10^8^ CFU/mL) were prepared in LB broth supplemented with 0.25% glucose, and 200 μL was dispensed into each well of a 96-well microplate. After incubation at 37 °C for 24 h, planktonic cells were removed and the biofilms were treated with 200 μL of KBU_SV1 at each MOI (corresponding to applied phage titers of 10^10^, 10^9^, 10^8^, 10^7^, and 10^6^ PFU/mL, respectively). Wells receiving 200 μL of PBS instead of phage were used as the untreated control. The plates were incubated at 37 °C for a further 24 h, washed three times with PBS, and the remaining biofilm biomass was measured by CV staining as described above.

To assess inhibition of biofilm formation, 20 μL of KBU_SV1 at each MOI (corresponding to applied phage titers of 10^11^, 10^10^, 10^9^, 10^8^, and 10^7^ PFU/mL, respectively) was added to 200 μL of bacterial suspension (10^8^ CFU/mL) prepared in LB broth supplemented with 0.25% glucose. For the untreated control, 20 μL of PBS was added to 200 μL of the bacterial suspension, and 20 μL of PBS added to 200 μL of sterile LB broth was used as the negative control. The plates were incubated overnight at 37 °C, and the biofilm biomass was measured as described above.

In both assays, the remaining biofilm biomass was expressed as a percentage of the untreated control, calculated as (mean OD₅₉₅ of the treated wells/mean OD₅₉₅ of the untreated control) × 100. Biofilm biomass reduction and inhibition of biofilm formation were calculated as 100 minus this value. All conditions were tested in triplicate.

### Phage DNA extraction and restriction analysis

Phage genomic DNA was extracted as described previously, with modifications (Uskudar-Guclu et al., 2024; Ali et al., 2023). Briefly, phage lysate (10^10^ PFU/mL) was centrifuged at 15,000 × g for 120 min at 4 °C to concentrate the phage particles. The phage preparation was resuspended in SM buffer (100 mM NaCl, 8 mM MgSO₄·7H₂O, 50 mM Tris–HCl [pH 7.5], and 0.01% [w/v] gelatin) and treated with DNase (Thermo Fisher Scientific, USA) at a final concentration of 1 μg/mL for 45 min at 37 °C to remove extracellular DNA. DNase was subsequently inactivated by incubation at 70 °C for 15 min. Proteinase K (Triogen, Türkiye) was then added to a final concentration of 20 μg/mL, followed by incubation at 56 °C for 45 min. Phage genomic DNA was purified by phenol-chloroform extraction followed by ethanol precipitation.

Restriction fragment length polymorphism (RFLP) analysis was performed using purified KBU_SV1 genomic DNA to assess its susceptibility to restriction endonucleases and to determine the resulting restriction profile. The endonucleases *EcoRI* and *HindIII* were selected as described in previous phage characterization studies (Ali et al., 2023; Damar Celik et al., 2024). The phage DNA was double-digested with these enzymes according to the manufacturer’s instructions. The resulting restriction fragments were separated by agarose gel electrophoresis and visualized under UV illumination.

### Sequence analysis and annotation of phage genome

Following extraction of phage genomic DNA, libraries were prepared using the Nextera DNA Prep Library Prep Kit (Illumina, San Diego, CA) and sequenced for 2 × 150 cycles on the Illumina Nextseq 500 (Illumina, USA) platform (Arkin et al., 2018). The quality of the raw data was assessed using FastQC v. 0.11.5 (Babraham Bioinformatics) and low-quality bases, primers and the remaining adapters were trimmed using Trimmomatic v. 0.32 (Bolger et al., 2014). High-quality reads were assembled using MEGAHIT v1.2.9 (Li et al., 2015). To validate the genome assembly, quality-filtered Illumina reads were mapped against the KBU_SV1 genome using Bowtie2 Galaxy version 2.5.5 (Langmead and Salzberg, 2012), and genome-wide sequencing depth and positional coverage were determined using SAMtools (Li et al., 2009). Genome completeness, contamination, and terminal repeat structure were further assessed using CheckV with the checkv-db-v1.5 database (Nayfach et al., 2021). Additionally, the presence of potential bacterial DNA contamination was assessed by searching the assembled genome against the database of 16S ribosomal RNA sequences (Bacteria and Archaea) using BLASTn (Camacho et al., 2009). The RASTtk v1.073 annotation pipeline was used for annotation (Brettin et al., 2015). BLASTp against NCBI’s Non-Redundant Protein Database was used to predict the functions of putative proteins. The RASTtk-based genome annotation was additionally evaluated using the phage-specific annotation pipeline Pharokka v1.14.6 as an independent assessment of predicted phage genes and their functional assignments (Bouras et al., 2023). The presence of tRNA was analyzed using tRNAscan-SE 2.0 (Lowe and Chan, 2016). The PhageLeads tool (Yukgehnaish et al., 2022) was also used to predict the lifestyle of KBU_SV1. In addition, the predicted lifestyle was independently evaluated using PhageAI v1.0.2 (Tynecki et al., 2020) based on the complete genome sequence. The genome annotation was also examined for canonical lysogeny-associated genes, including integrases, excisionases, and phage repressors. The presence of antibiotic resistance and virulence-associated genes in the phage genome was investigated using VirulenceFinder 2.0 and ResFinder 4.1 (Bortolaia et al., 2020; Malberg Tetzschner et al., 2020). As an additional genome-wide screening approach, the genome was analyzed using the Resistance Gene Identifier (RGI) against the Comprehensive Antibiotic Resistance Database (CARD v4.0.2) and the Virulence Factor Database (VFDB) (Raghuvanshi and Dwivedi, 2026; Edalatmand et al., 2026). Only “Perfect” and “Strict” matches were considered in the CARD/RGI analysis.

To identify closely related phage genomes, the complete genome sequence of KBU_SV1 was searched against the NCBI nucleotide database using BLASTn (Camacho et al., 2009). Closely related complete phage genomes were selected for subsequent comparative genomic analyses based on nucleotide similarity and query coverage. Easyfig v2.2.5 was used to compare phage genomes (Sullivan et al., 2011). Linkages were filtered with a minimum alignment length of 1 kb, E-value ≤1e−3, and identity shading of 22–100%. Pairwise intergenomic similarities between KBU_SV1 and closely related complete bacteriophage genomes identified by BLASTn were calculated using the Virus Intergenomic Distance Calculator (VIRIDIC) with default settings (Moraru et al., 2020). Intergenomic similarity values of ≥95% and ≥70% were considered indicative of species- and genus-level genomic clustering, respectively. The resulting similarity matrix and heatmap were used to evaluate genomic relationships among KBU_SV1 and related phages.

### Phylogenetic analysis

The phylogenetic tree was constructed using MEGA version 11.0 with a neighbor-joining method employing a Poisson model with 1,000 bootstrap repeats, based on the amino acid sequences of the phages’ large terminase subunit (Tamura et al., 2021). Additionally, VipTree 4.1 was used to construct the viral proteomic tree (Nishimura et al., 2017). The proteomes of KBU_SV1 and 15 highly similar phage genomes were compared to reference genomes containing prokaryotic double-stranded DNA viruses in the database using tBLASTx. Furthermore, the taxonomic position of KBU_SV1 was independently assessed using taxMyPhage v3.3.6 (Millard et al., 2025), which compares the queried dsDNA phage genomes to reference genomes classified by ICTV using genome-based genus and species-level classification criteria. Taxonomic classifications were interpreted according to the current ICTV taxonomy.

### Assessing the antimicrobial activity of phages using G*alleria mellonella* larvae

*Galleria mellonella* larvae (Lepidoptera: Pyralidae) at the final instar stage (200–300 mg) were used as an *in vivo* infection model to evaluate the activity of KBU_SV1 against *E. coli*, as described previously with modifications (Chen and Keddie, 2021). Larvae were reared at 27 ± 2 °C in glass jars on an artificial diet consisting of ground wheat and maize (22% each), beeswax (17.5%), honey, glycerol and milk powder (11% each), and yeast extract (5.5%). Only larvae that were uniformly cream-coloured, showed no signs of melanization and responded to physical stimulation were used.

For bacterial preparation, *E. coli* ATCC 25922 was grown overnight in 10 mL of LB broth at 37 °C. The culture was adjusted to 10^7^ CFU/mL, centrifuged at 15,000 × *g* for 5 min at 4 °C, washed twice with sterile phosphate-buffered saline (PBS) and resuspended in an equal volume of PBS to restore the initial density. To prepare the phage for *in vivo* injection and to minimize soluble lysate-derived contaminants such as bacterial proteins and endotoxins/LPS, the KBU_SV1 phage filtrate was subjected to high-speed centrifugation at 15,000 × *g* for 120 min at 4 °C to pellet the phage particles (Bonilla et al., 2016). The supernatant containing soluble bacterial components was discarded, and the pellet was resuspended in sterile PBS, filtered through a 0.22-μm membrane, and adjusted to 10^9^ PFU/mL.

Injections were performed into the haemocoel through the second left proleg using a Hamilton syringe (26–30G), as described previously (Tsai et al., 2016). For infection, 10 μL of the bacterial suspension was injected, corresponding to approximately 10^5^ CFU per larva. This challenge dose was determined based on preliminary dose-finding experiments (data not shown) as the minimum inoculum required to consistently cause 100% larval mortality within 24 h. Larvae in the phage-only control and treatment groups received 10 μL of the phage preparation, corresponding to approximately 1 × 10^7^ PFU per larva and, in the treatment group, to a multiplicity of infection of approximately 100. The experimental groups were: (I) a phage-only control receiving phage alone, (II) a treatment group receiving phage 1 h after bacterial injection, (III) a bacteria-only infection control, and (IV) a mock-injection control receiving 10 μL of sterile PBS to evaluate potential mortality from the injection procedure itself. Each group contained 10 larvae and the experiment was performed independently twice, giving 20 larvae per group in total. Larvae were incubated at 37 °C and examined at 6-h intervals for 48 h. Larvae were scored as dead when they were fully melanized and unresponsive to physical stimulation.

### Statistical analysis

The bacterial growth reduction assay, the pH and thermal stability assays, and the biofilm reduction and prevention assays were performed in triplicate, and data are presented as the mean ± standard deviation (SD). Statistical comparisons were performed using one-way ANOVA followed by Tukey’s multiple comparisons test for the pH and thermal stability assays. The analyses included pH 4, 6, 7, 8, 10, and 12 for pH stability, and 4, 23 and 37 °C for thermal stability. Conditions at which no plaques were detected (pH 2 and 65 °C) were excluded from the analyses of log-transformed titers.

For the *Galleria mellonella* infection model, two independent experiments were performed with 10 larvae per group. The two experiments yielded identical survival patterns, and the data were therefore pooled for analysis (*n* = 20 per group), with each larva treated as an independent observation. Kaplan–Meier survival curves were generated and compared using the log-rank (Mantel–Cox) test in GraphPad Prism software (version 9.0; GraphPad Software, San Diego, CA, USA). A *p*-value < 0.05 was considered statistically significant.

## Results

### Isolation, purification and morphological characterization of KBU_SV1 phage

#### Isolation, purification and plaque morphology

A bacteriophage capable of infecting the indicator strain *E. coli* ATCC 25922 was successfully isolated from sewage samples collected in Karabük, Türkiye, and was designated KBU_SV1. Following enrichment, the phage was purified by four consecutive rounds of single-plaque isolation. Before purification, plaques of differing sizes and appearance were observed on the bacterial lawn, indicating a heterogeneous phage preparation (Figure 1A). After purification, a homogeneous population of uniform plaques was obtained (Figure 1B). KBU_SV1 produced circular, clear plaques with well-defined edges after overnight incubation at 37 °C. The titre of the purified phage lysate was determined by enumerating plaques on the indicator strain using the double-agar layer method and was approximately 10^8^ PFU/mL.

**Figure 1:**
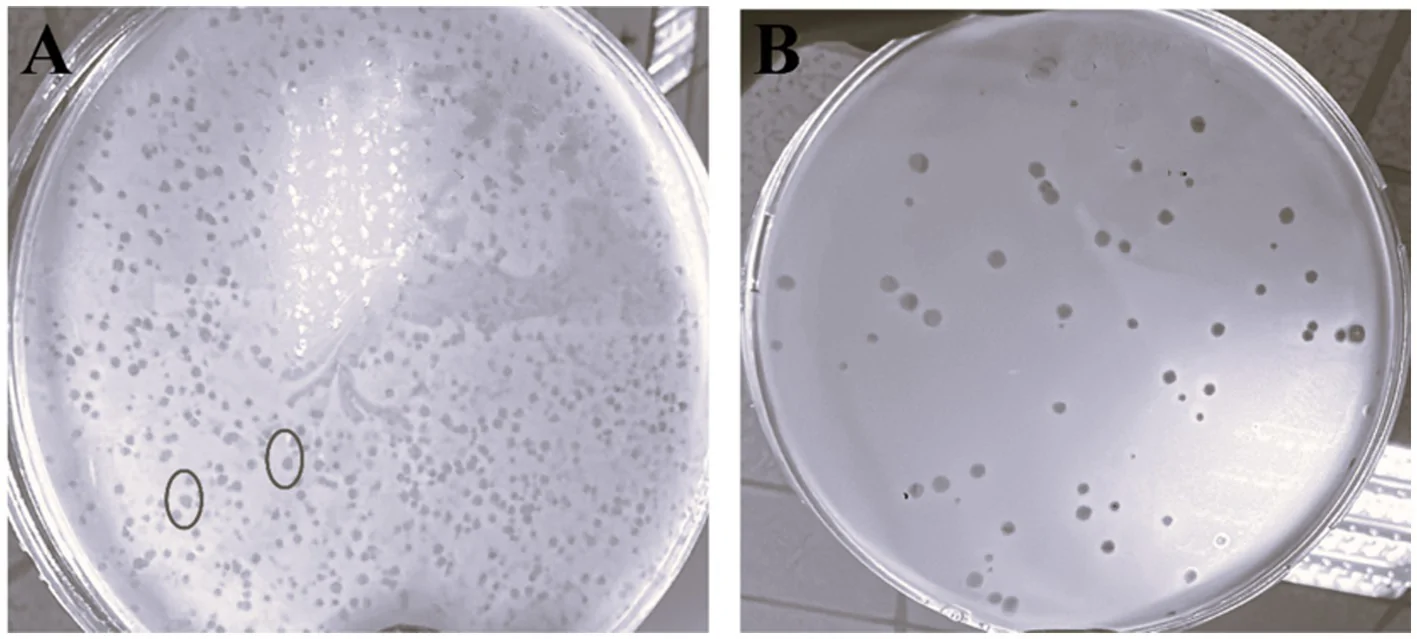
Plaque morphology of KBU_SV1 on *E. coli* ATCC 25922 using the double-agar layer method. **(A)** Plaques observed before purification. **(B)** Uniform plaques obtained after four rounds of plaque purification.

#### Transmission electron microscope (TEM) analysis

Transmission electron microscopy of negatively stained preparations revealed that KBU_SV1 has a polyhedral head and a long, flexible, non-contractile tail (Figure 2). In the representative virion analyzed, the head diameter was approximately 45 nm and the tail length approximately 176 nm, giving a total particle length of about 220 nm and a tail-to-head ratio of approximately 3.9. No contractile sheath or baseplate structure was observed. This morphology corresponds to the siphovirus morphotype within the class *Caudoviricetes*.

**Figure 2:**
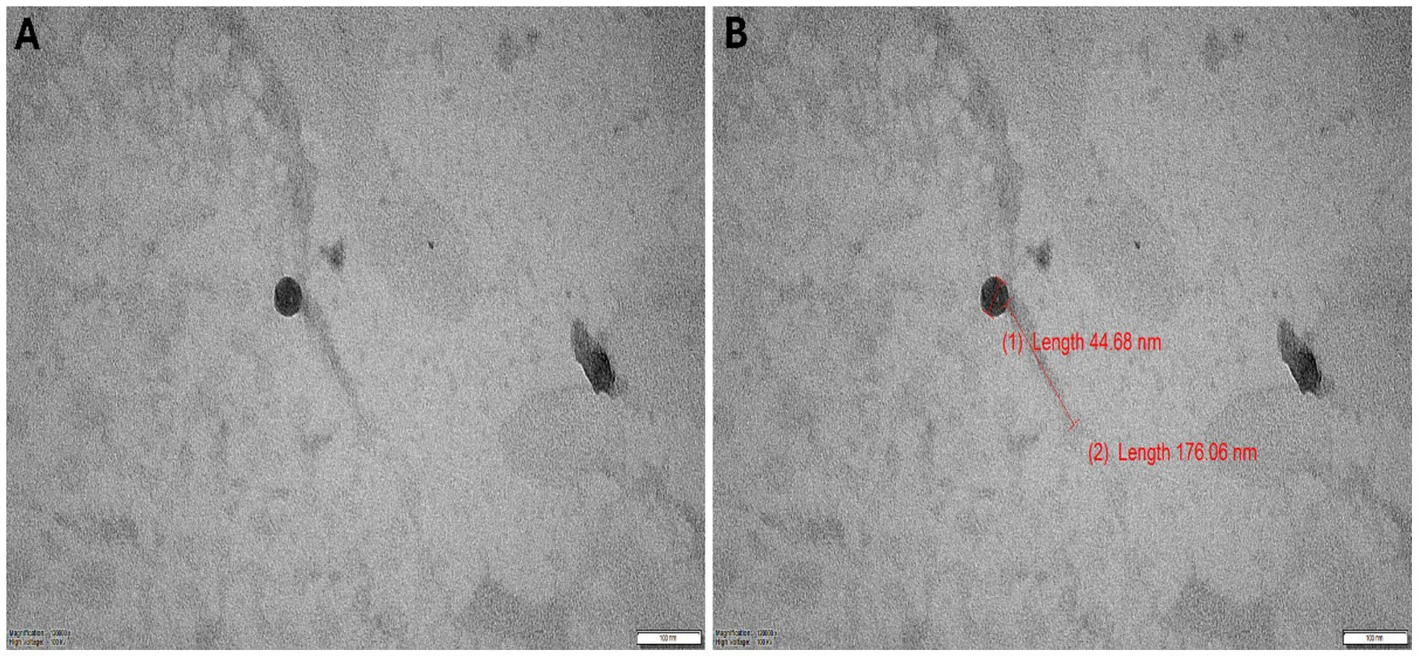
Transmission electron micrographs of *Escherichia* phage KBU_SV1 negatively stained with 2% uranyl acetate. **(A)** Unlabeled image showing the polyhedral head and long, flexible, non-contractile tail. **(B)** The same field with the measured head diameter (1) and tail length (2) indicated. Scale bars = 100 nm.

### Host range analysis of KBU_SV1 phage

The host range of KBU_SV1 was evaluated against a panel of 106 bacterial strains comprising clinical isolates of *E. coli*, *K. pneumoniae* and *E. faecalis* together with six reference strains (Supplementary Table S1). KBU_SV1 exhibited spot clearance against 16 of the 106 strains tested, corresponding to an HRI of 0.15.

Spot clearance was restricted to members of the family *Enterobacteriaceae* and was observed most frequently among carbapenem-resistant *E. coli*. Clear zones of lysis were detected on 7 of the 20 CREC isolates (35%), 4 of the 20 CSEC isolates (20%) and 4 of the 40 CRKP isolates (10%), as well as on the propagation host *E. coli* ATCC 25922. Within the *E. coli* isolates, spot clearance was therefore recorded more frequently among carbapenem-resistant than among carbapenem-susceptible isolates, whereas the proportion of susceptible strains was lowest among the CRKP isolates. The CREC and CRKP isolates showing clearance by KBU_SV1 were multidrug-resistant clinical strains carrying carbapenemase and extended-spectrum β-lactamase genes (Supplementary Table S1).

No spot clearance was detected against any of the 20 clinical *E. faecalis* isolates, nor against the *S. aureus* ATCC 29213, *E. faecalis* ATCC 29212, *P. aeruginosa* ATCC 27853, *A. baumannii* ATCC 19606 and *K. pneumoniae* ATCC 700603 reference strains.

### Bacterial reduction by KBU_SV1 phage at different MOIs

The antibacterial activity of KBU_SV1 was evaluated by monitoring bacterial growth at different multiplicities of infection (MOIs) over a 24-h incubation period (Figure 3). In the untreated control, growth entered the exponential phase after 4 h and OD₅₉₅ increased continuously throughout the incubation, reaching 1.70 at 24 h. In contrast, OD₅₉₅ values in all phage-treated cultures remained below 0.10 during the first 14 h of incubation at every MOI tested, corresponding to a reduction of at least 93% relative to the control at 14 h.

**Figure 3:**
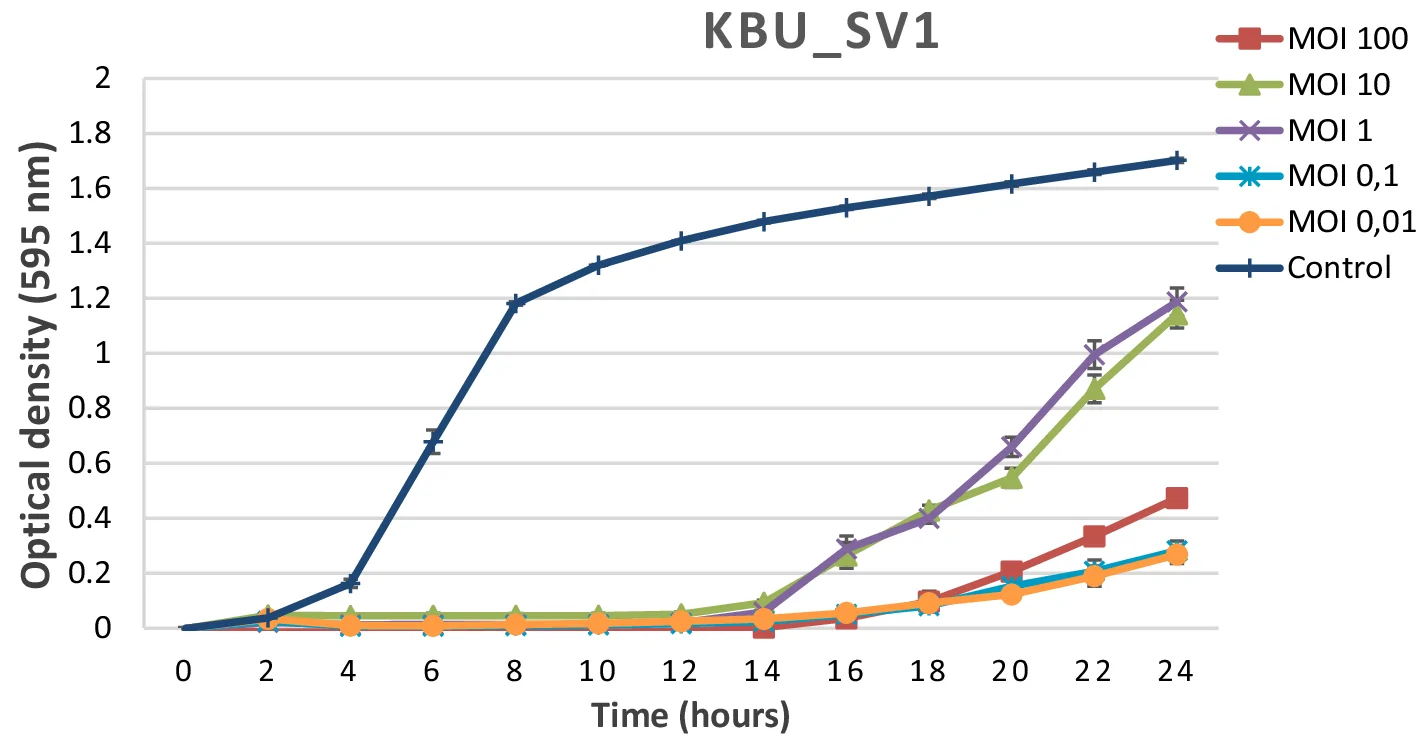
Growth of *E. coli* ATCC 25922 in the presence of KBU_SV1 at different multiplicities of infection (MOIs of 100, 10, 1, 0.1, and 0.01) and in the untreated growth control. OD₅₉₅ was measured every 2 h for 24 h. Data are presented as the mean ± SD of three independent experiments.

Bacterial regrowth was subsequently observed under all conditions, but its onset and extent differed between MOIs. At MOIs of 10 and 1, OD₅₉₅ increased markedly from 16 h onwards and reached 1.14 and 1.19 at 24 h, corresponding to reductions of only 33 and 30% relative to the control. At MOIs of 100, 0.1 and 0.01, OD₅₉₅ remained below 0.10 for up to 18 h and increased more gradually thereafter, reaching 0.47, 0.28 and 0.27 at 24 h, corresponding to reductions of 72%, 83 and 84% relative to the control.

Overall, KBU_SV1 suppressed bacterial growth across the entire range of MOIs tested during the first 14 h of incubation. At 24 h, the mean OD₅₉₅ values were lower at MOIs of 100, 0.1 and 0.01 than at MOIs of 10 and 1, indicating an apparent non-monotonic relationship between the initial MOI and the subsequent growth response.

### Stability of KBU_SV1 phage at different pH and temperatures

KBU_SV1 retained its infectivity across a broad pH range. After 60 min of incubation at 4 °C, residual titres at pH 4, 6, 7, 8, 10 and 12 remained at approximately 10^8^ PFU/mL. However, titres at pH 4, 6 and 12 were significantly lower than those at the pH 7 reference condition (*p* < 0.01; Figure 4A). The mean titre at pH 7 was 8.24 ± 0.06 log₁₀ PFU/mL, whereas the mean titres at pH 4, 6 and 12 were 7.99 ± 0.02, 7.99 ± 0.03 and 7.99 ± 0.05 log₁₀ PFU/mL, respectively. These corresponded to reductions of approximately 0.26, 0.25 and 0.25 log₁₀ PFU/mL relative to pH 7, respectively. No statistically significant differences were detected between pH 7 and pH 8 or 10 (*p* > 0.1). In contrast, no plaques were recovered after incubation at pH 2.

**Figure 4:**
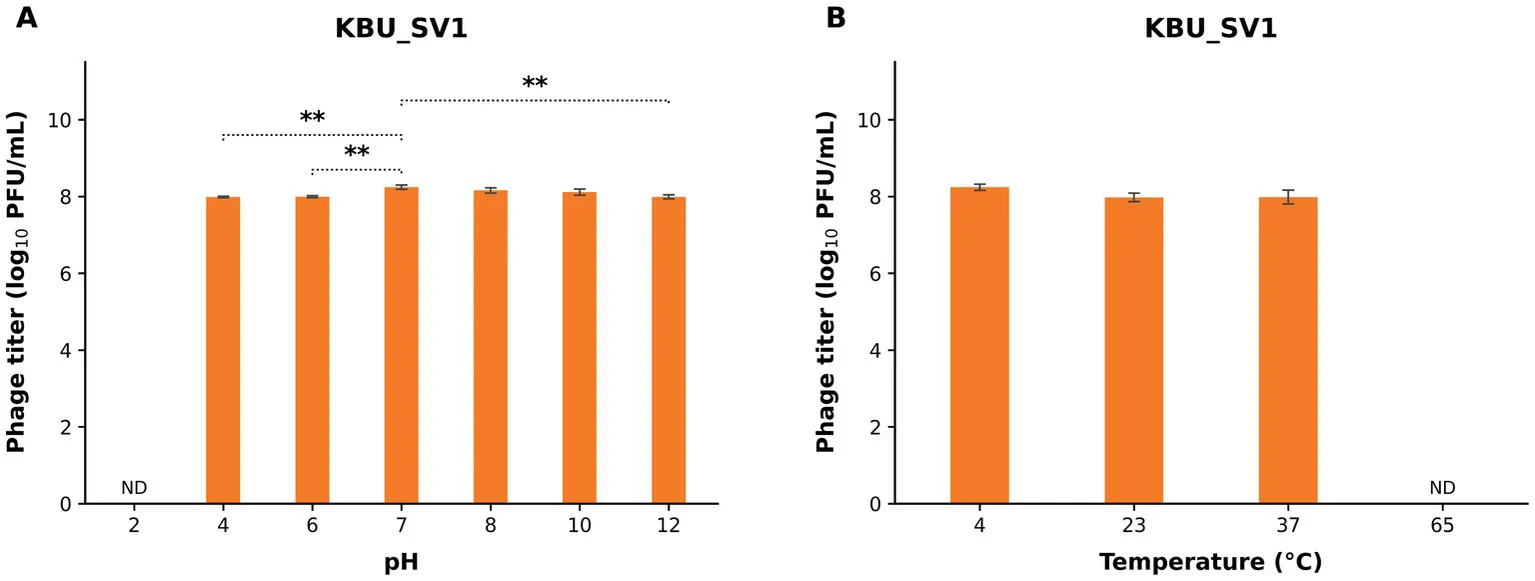
Stability of KBU_SV1 under different pH and temperature conditions. **(A)** Residual phage titres (log₁₀ PFU/mL) after 60 min of incubation at 4 °C in SM buffer adjusted to pH 2–12. **(B)** Residual phage titres (log₁₀ PFU/mL) after 60 min of incubation at 4, 23, 37, and 65 °C. Bars represent the mean ± SD (*n* = 3). The two assays were analyzed separately using one-way ANOVA followed by Tukey’s HSD test. In **(A)**, brackets indicate significant comparisons with pH 7 (**p-value <0.01). No significant differences were detected among 4, 23, and 37 °C in **(B)**. ND indicates that no plaques were detected.

KBU_SV1 was similarly stable over the temperature range tested up to physiological temperature. Residual titres after 60 min of incubation at 4 °C, 23 °C and 37 °C remained at approximately 10^8^ PFU/mL, with mean values of 8.24 ± 0.08, 7.98 ± 0.11 and 7.98 ± 0.18 log₁₀ PFU/mL, respectively. No statistically significant differences were detected among these temperatures (*p* > 0.1, Figure 4B). No plaques were recovered after incubation at 65 °C, indicating loss of detectable infectivity under these conditions.

### Antibiofilm activity of KBU_SV1 phage

Treatment of established biofilms of *E. coli* ATCC 25922 with KBU_SV1 left 13.96–15.48% of the biofilm biomass of the untreated control at all tested MOIs, corresponding to a biomass reduction of 84.52–86.04% (Figure 5A). For the carbapenem-resistant clinical isolate Ec59, the remaining biofilm biomass ranged from 45.85 to 77.66%, corresponding to a biomass reduction of 22.34–54.15% (Figure 5B).

**Figure 5:**
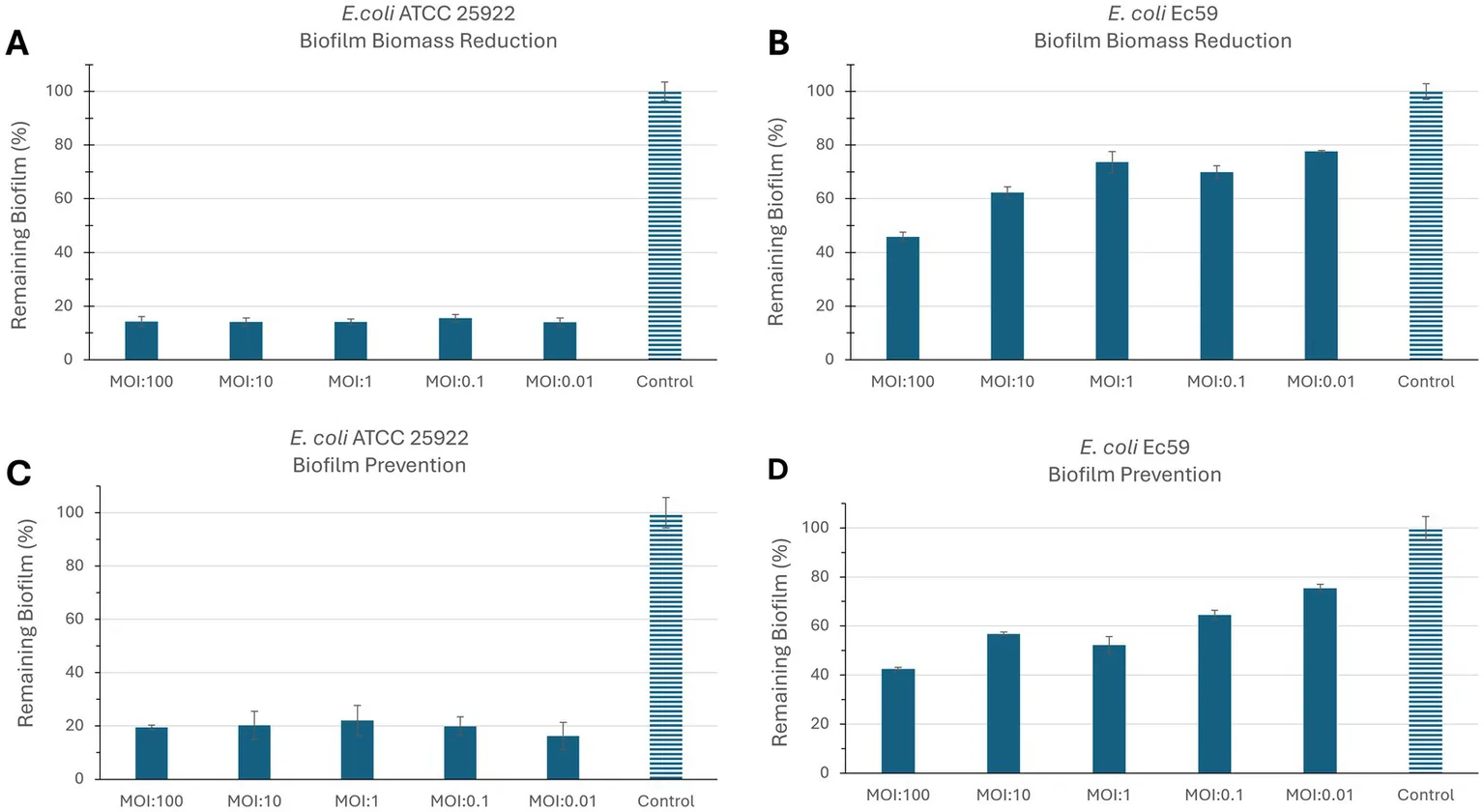
Antibiofilm activity of KBU_SV1 against *E. coli* ATCC 25922 and the carbapenem-resistant clinical isolate Ec59. **(A,B)** Remaining biofilm biomass after treatment of established biofilms with KBU_SV1 at MOIs of 100, 10, 1, 0.1, and 0.01. **(C,D)** Biofilm biomass formed in the presence of KBU_SV1 at the same MOIs. Biofilm biomass was quantified by crystal violet staining and is expressed as a percentage of the untreated control, which was set to 100%. Bars represent the mean ± SD of three independent experiments.

In the biofilm inhibition assay, the biomass formed by *E. coli* ATCC 25922 in the presence of KBU_SV1 ranged from 16.27 to 22.04% of the untreated control, corresponding to 77.96–83.73% inhibition of biofilm formation (Figure 5C). For Ec59, the corresponding values ranged from 42.50 to 75.39%, corresponding to 24.61–57.50% inhibition (Figure 5D).

In both assays, the remaining biofilm biomass was lower for *E. coli* ATCC 25922 than for Ec59. For ATCC 25922, comparable residual levels were obtained across all tested MOIs, whereas for Ec59 the residual biomass was lowest at MOI 100 (45.85% in the treatment assay and 42.50% in the inhibition assay) and increased as the MOI decreased, indicating an MOI-dependent effect against the clinical isolate that was not apparent for the reference strain.

### *In vivo* activity of KBU_SV1 in the G*alleria mellonella* infection model

The *in vivo* activity of the KBU_SV1 phage was evaluated using the *Galleria mellonella* infection model with *E. coli* ATCC 25922. No mortality was observed in the phage-only control group or the PBS-only mock-injection control group during the 48-h observation period. In contrast, larvae infected with bacteria alone exhibited rapid mortality, with 40% of the larvae dying at 12 h post-infection and the remaining 60% dying by 18 h, resulting in complete mortality of the bacteria-only control group (Figure 6). Phage treatment markedly delayed larval mortality and altered the progression of infection. In the phage-treated group, mortality occurred later and was distributed over a longer period, with 20% mortality at 18 h, 40% at 24 h, 20% at 42 h, and 20% at 48 h post-infection. Consequently, 40% of the larvae remained alive at 24 h following phage administration. Kaplan–Meier survival analysis demonstrated a significant delay in larval mortality in the phage-treated group compared with the bacteria-only control group. Statistical comparison of the survival curves using the log-rank (Mantel–Cox) test confirmed a significant difference between the groups (χ^2^ = 27.96, df = 1, *p* = 1.2 × 10^−7^) (Figure 6).

**Figure 6:**
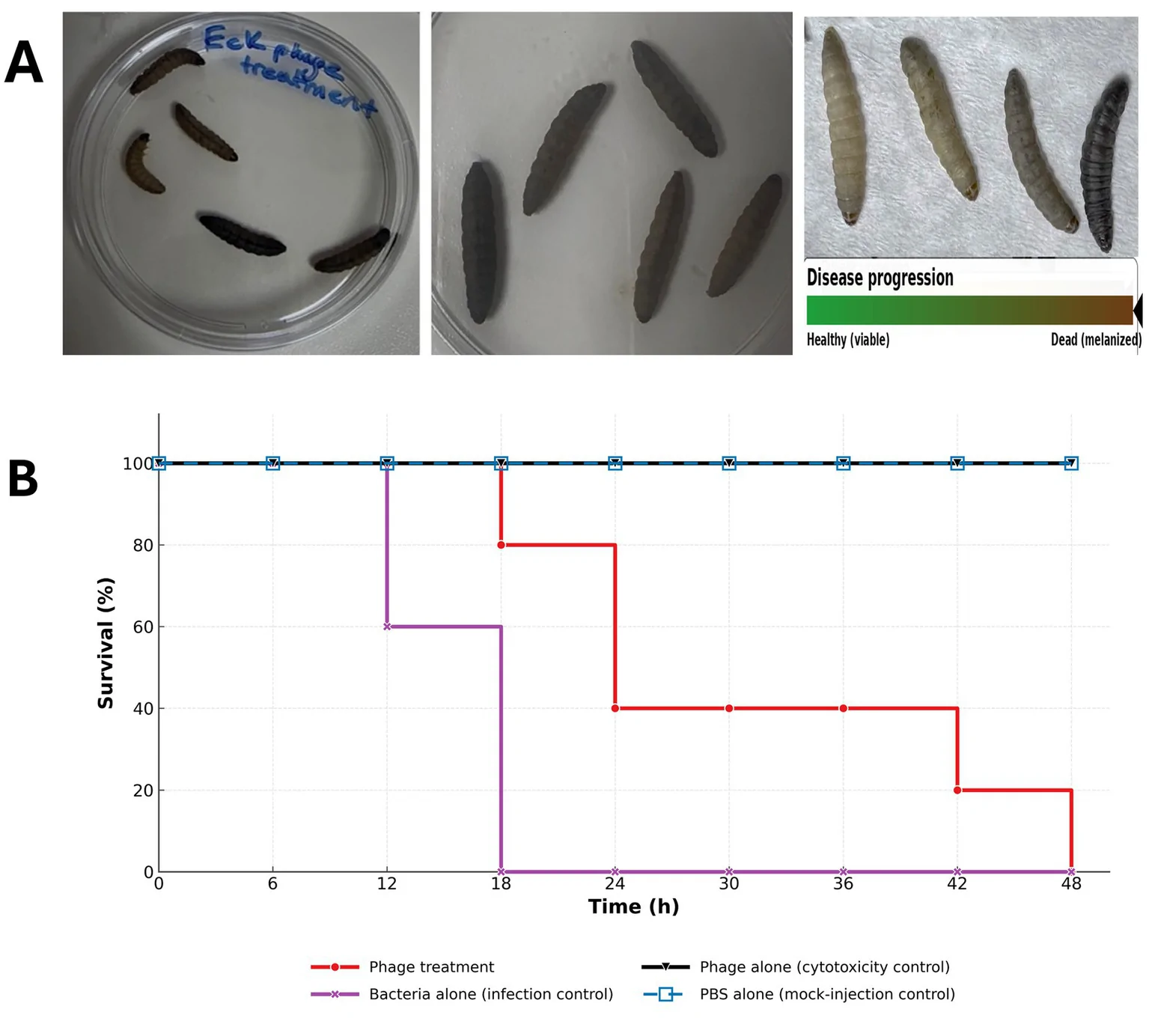
Survival of *Galleria mellonella* larvae infected with *Escherichia coli* ATCC 25922 following KBU_SV1 treatment. **(A)** Representative larvae from the phage-treated group (left) and the infection control (middle), and the melanization scale (right), ranging from healthy (cream-colored) to dead (fully melanized). **(B)** Kaplan–Meier survival curves of larvae receiving phage alone (cytotoxicity control), sterile PBS alone (mock-injection control)*, E. coli* ATCC 25922 alone (infection control), or *E. coli* ATCC 25922 followed by KBU_SV1 (≈1 × 10^7^ PFU per larva; MOI ≈ 100; treatment group). Data are pooled from two independent experiments with 10 larvae per group, which yielded identical survival patterns (*n* = 20 per group). Curves were compared using the log-rank (Mantel–Cox) test (*p* < 0.0001).

### Restriction profile of KBU_SV1 genomic DNA

Restriction analysis of KBU_SV1 genomic DNA following double digestion with *EcoRI* and *HindIII* produced multiple DNA fragments ranging approximately from 500 to 10,000 bp (Supplementary Figure S1). This analysis was initially performed as a preliminary step prior to whole-genome sequencing to confirm the double-stranded nature of the extracted DNA and its susceptibility to these specific endonucleases, providing a basic physical fingerprint.

### Genomic characterization of *Escherichia* phage KBU_SV1

Genomic DNA of *Escherichia* phage KBU_SV1 was sequenced using next-generation sequencing (NGS). The paired-end Illumina sequencing generated 4,057,836 read pairs with an average read length of approximately 150 bp. Of these, 3,989,842 reads (98.3%) passed quality-control filtering, and 3,642,452 reads (91.3% of the quality-filtered reads) were retained for downstream analysis. Read-mapping analysis provided strong support for the integrity of the 45,190-bp KBU_SV1 genome assembly. Mapping of the paired-end Illumina reads against the assembled genome resulted in an overall alignment rate of 92.5%. All 45,190 genomic positions were supported by sequencing reads, corresponding to 100% genome coverage, with a mean sequencing depth of 24,181.9 × and no zero-coverage positions. CheckV independently classified the KBU_SV1 genome as complete, with 100% estimated completeness and 0% contamination, and assigned it to the MIUViG high-quality category. CheckV also identified a 272-bp direct terminal repeat (DTR) with high confidence at both termini of the genome.

The entire genome of *Escherichia* phage KBU_SV1 was sequenced (Figure 7). The phage has a 45,190-bp double-stranded DNA genome with a G + C content of 53.56%. In total, the KBU_SV1 genome encodes 71 open reading frames (ORFs), 33 of which (46.4%) have been assigned hypothetical functions. Five different modules were identified in the genome: DNA/RNA replication, DNA metabolism, structural proteins, assembly module, and lysis module. The lysis module contains genes encoding endolysin, holin, and spanin. The phage assembly module contains genes for head morphogenesis and terminase. The structural module contains genes encoding tail fiber proteins, major and minor head proteins, head closure Hc1, Neck1 protein, tail terminator, and tail length tape measure proteins. The DNA replication module includes DNA polymerase, RNA polymerase, DNA ligase, helicase, and restriction alleviation protein. The DNA metabolism module consists of DNA methyltransferase, endonuclease, and exonuclease genes. One ORF was initially annotated as a putative transposase by the automated annotation pipeline. However, InterProScan analysis detected no transposase-related domain in this protein, and BLASTp searches returned matches only to hypothetical proteins. This ORF was therefore considered a hypothetical protein rather than a transposase, and no transposase or other mobile genetic element–associated gene was identified in the KBU_SV1 genome. Other proteins encoded in the genome are referred to as hypothetical proteins.

**Figure 7:**
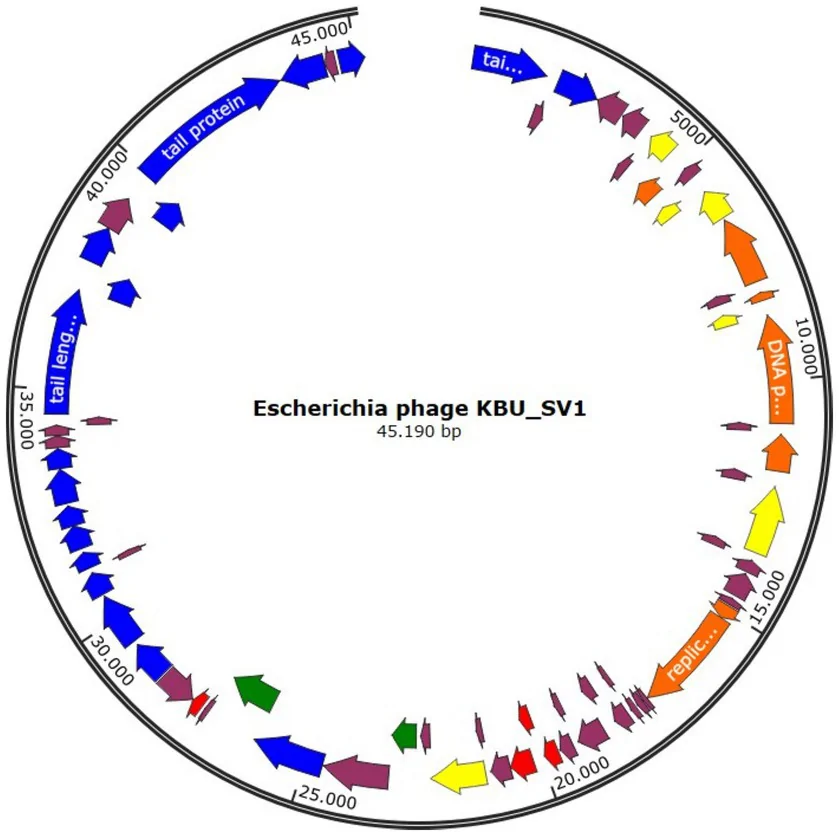
Genome map of *Escherichia* phage KBU_SV1, showing the 71 predicted open reading frames (ORFs) and a G + C content of 53.56%. Arrows indicate the positions and orientations of the ORFs, colored according to functional group: DNA replication, orange; DNA metabolism, yellow; assembly, green; lysis, red; structural, blue; hypothetical, purple. Putative functions were predicted using BLASTp searches against the NCBI non-redundant protein database and InterProScan domain analysis. The genome is displayed in circular format for visualization purposes. The map was generated with SnapGene Viewer 5.3.2.

No tRNA genes were identified in the KBU_SV1 genome. Screening against the bacterial and archaeal 16S rRNA database revealed no significant matches, providing no evidence of detectable bacterial DNA contamination by this approach, consistent with the 0% contamination estimated by CheckV. Genome-based lifestyle assessment supported a predicted virulent (lytic) lifestyle for KBU_SV1. PhageAI classified KBU_SV1 as virulent, consistent with the PhageLeads assessment and with the absence of canonical lysogeny-associated genes, including integrases, excisionases, and phage repressors, in the genome annotation. No known antimicrobial resistance or virulence-associated genes were detected in the KBU_SV1 genome using ResFinder and VirulenceFinder. Additional screening against CARD/RGI identified no Perfect or Strict antimicrobial resistance hits, while VFDB analysis detected no known virulence factors. Collectively, these complementary database searches provided no evidence of known antimicrobial resistance or virulence determinants in the KBU_SV1 genome.

#### Comparative genomic analysis

Similarities between *Escherichia* phage KBU_SV1 and other phage genomes were examined using BLASTn. BLASTn analysis of the complete KBU_SV1 genome against the NCBI nucleotide database revealed high nucleotide similarity with several related phages. Among the closest matches, *Escherichia* phage EPIMAM03 (PX514237.1) showed 99% query coverage and 99.12% nucleotide identity, whereas *Escherichia* phage Ioannina (OQ589852.1) exhibited 96% query coverage and 99.64% nucleotide identity. Additional highly similar genomes were identified, including *Dhillonvirus* LM40P1 and *Escherichia* phage BorMax. These findings indicated a close genomic relationship between KBU_SV1 and the identified phages. A representative genome comparison between *Escherichia* phage KBU_SV1 and selected closely related reference genomes (EPIMAM03 and Ioannina) is shown in Figure 8. To further resolve the genome-based relationship of KBU_SV1 with closely related phages, pairwise intergenomic similarities were calculated using VIRIDIC (Figure 9). KBU_SV1 exhibited the highest intergenomic similarity with *Dhillonvirus* LM40P1 (OZ035780.1; 98.7%), followed by *Escherichia* phage EPIMAM03 (PX514237.1; 98.6%), *Dhillonvirus* LM40P1 (OZ035746.1; 98.5%), *Dhillonvirus* LM40P1 (OZ035811.1; 97.4%), *Escherichia* phage BorMax (PX586628.1; 96.2%), and *Escherichia* phage Ioannina (OQ589852.1; 95.3%). The three LM40P1 entries represent distinct genome assemblies deposited in NCBI under different accession numbers and reflect sequence differences among these records. In contrast, intergenomic similarities with *Escherichia* phage KCP_038, *Escherichia* phage Ecol_81, *Escherichia* phage vB_EcoS_WFI, and *Sodalis* phage SO1 were below the 95% species-level threshold. All compared genomes shared intergenomic similarities above the 70% genus-level threshold (72.6–98.7%), consistent with their assignment to a single genus, whereas values ≥95% were restricted to KBU_SV1, LM40P1, EPIMAM03, BorMax and Ioannina. Thus, KBU_SV1 belongs to an existing species-level genomic cluster rather than representing a distinct bacteriophage species.

**Figure 8:**
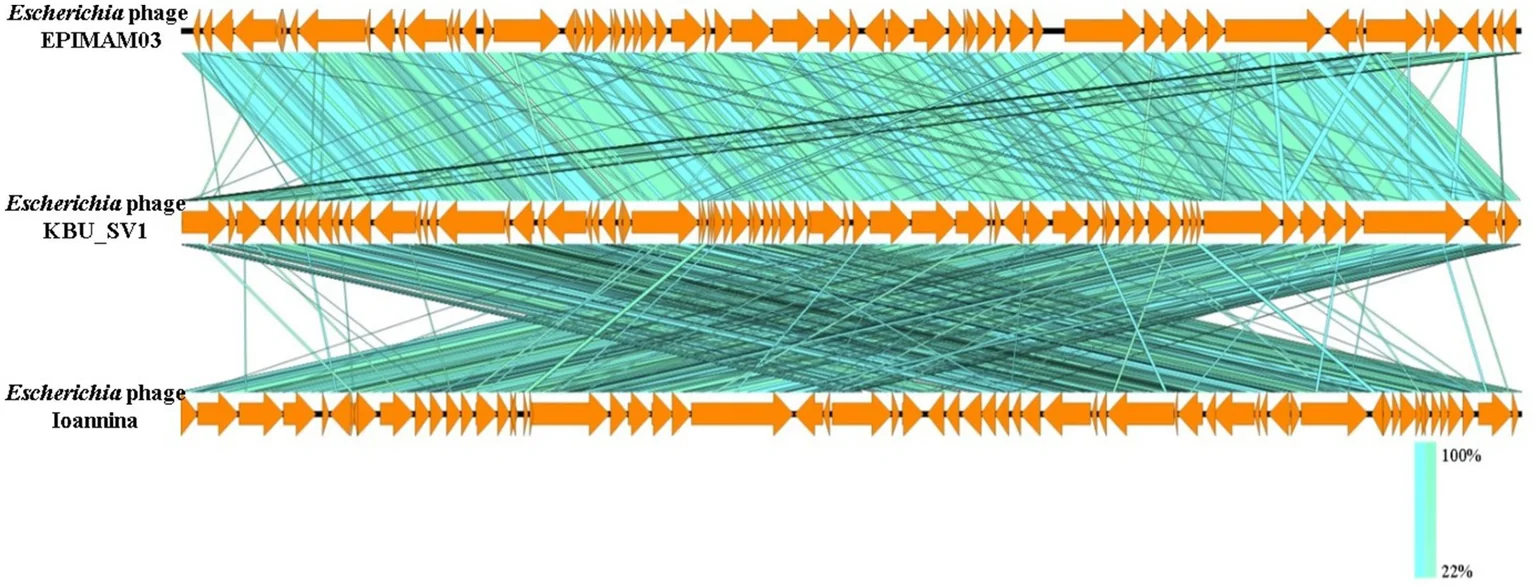
Comparative genomic analysis of *Escherichia* phage KBU_SV1 and closely related reference phages, visualized with Easyfig using BLASTn. Alignment of KBU_SV1 with *Escherichia* phage EPIMAM03 and *Escherichia* phage Ioannina revealed extensive nucleotide similarity across the full length of the genomes. Colored links indicate regions of homology, with shading intensity corresponding to the level of nucleotide identity.

**Figure 9:**
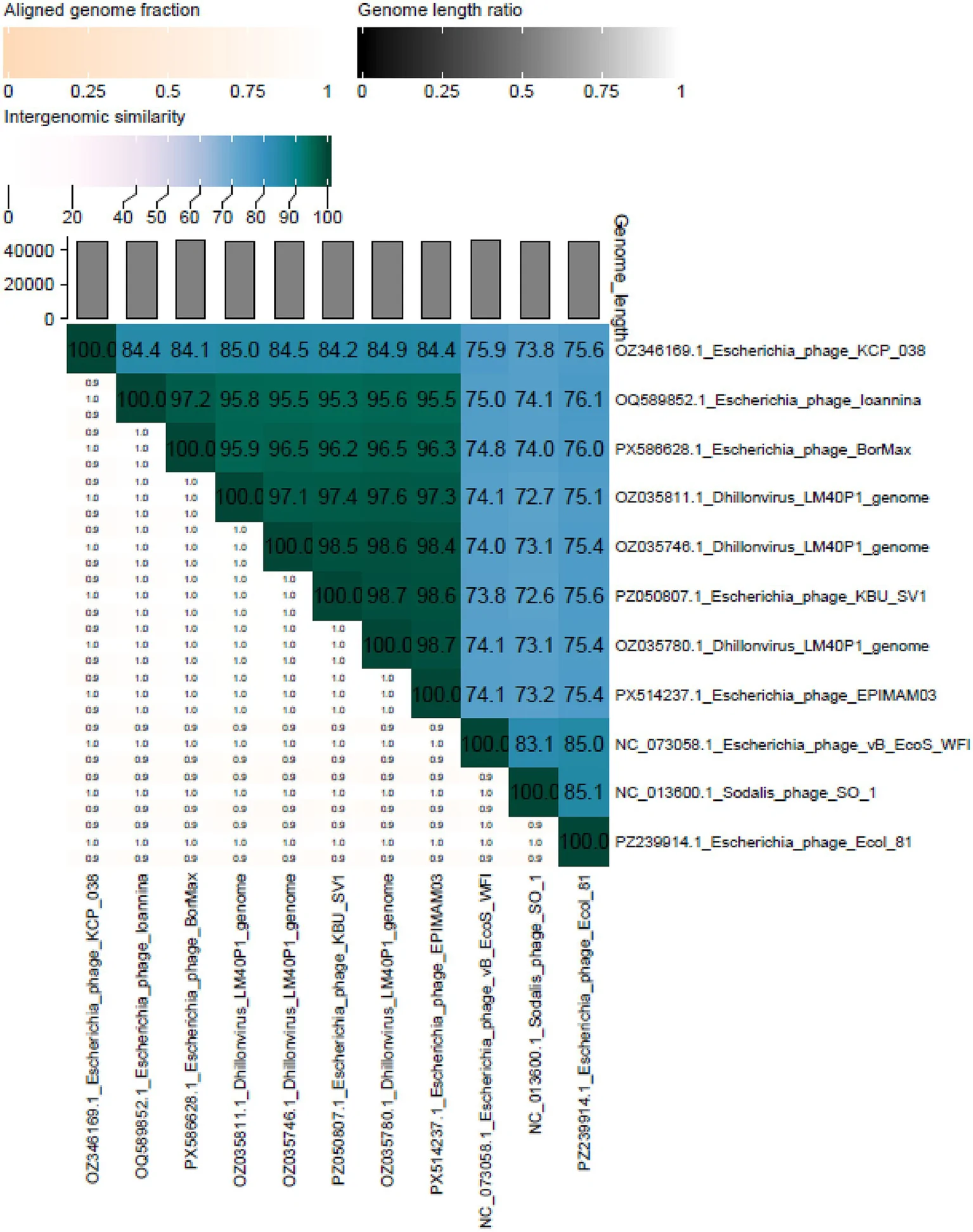
Intergenomic similarity analysis of *Escherichia* phage KBU_SV1 and closely related bacteriophages using VIRIDIC. Pairwise intergenomic similarity values (%) are shown in the upper triangular matrix, whereas aligned genome fractions and genome-length ratios are displayed in the lower triangular matrix. Genomes were hierarchically clustered based on intergenomic similarities. KBU_SV1 showed >95% intergenomic similarity with several closely related phages, supporting its placement within the same species-level genomic cluster.

#### Taxonomic and phylogenetic analysis of *Escherichia* phage KBU_SV1

Taxonomic and phylogenetic analyses of *Escherichia* phage KBU_SV1 were performed using representative enterobacterial phages. Two complementary approaches were employed for phylogenetic reconstruction. First, the amino acid sequence of the large terminase subunit (TerL) of KBU_SV1 was aligned with homologous sequences retrieved from NCBI using MUSCLE, and a neighbor-joining phylogenetic tree was constructed (Figure 10). The TerL-based phylogeny showed that KBU_SV1 formed a well-supported cluster with *Escherichia* phage SP1 (PV610739.1) and *Escherichia* phage EPIMAM03 (PX514237.1). Notably, KBU_SV1 clustered most closely with EPIMAM03, supported by a bootstrap value of 97, while the broader clade including SP1 was supported by a bootstrap value of 100. Other phages included in the analysis, such as *Serratia* phage Scapp, *Edwardsiella* phage eiAU, and *Escherichia* phage Ioannina, were resolved on more distant branches, whereas *Klebsiella* phage 37P2 appeared as a more divergent lineage.

**Figure 10:**
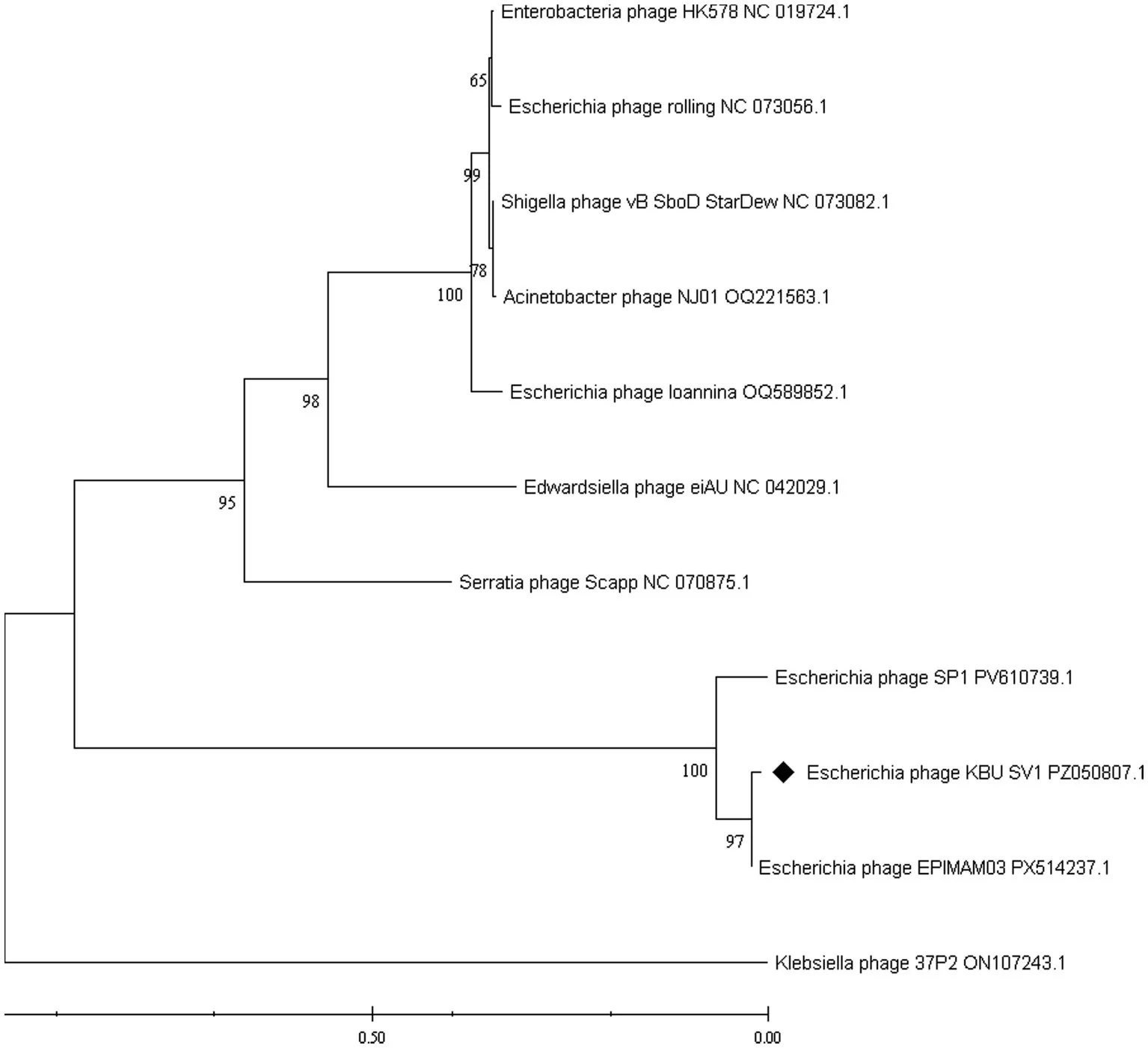
Neighbor-Joining phylogenetic tree based on the amino acid sequence of the large terminase subunit (TerL), showing the evolutionary relationship of *Escherichia* phage KBU_SV1 with representative bacteriophages. KBU_SV1 clustered most closely with *Escherichia* phage EPIMAM03 (bootstrap support, 97%) and formed a strongly supported clade with *Escherichia* phage SP1. Bootstrap values are indicated at the nodes. KBU_SV1 is indicated by a black diamond. The scale bar represents the number of amino acid substitutions per site.

Second, genome-wide proteomic comparisons were performed using VipTree to construct a proteomic tree based on whole-genome similarities (Figure 11). Consistent with the TerL-based phylogeny, KBU_SV1 was positioned within a cluster of closely related *Escherichia* phages, showing high similarity to phages such as *Escherichia* phage KarrBarth, *Escherichia* phage vB_EcoS_WFI, and *Escherichia* phage vB_EcoS-WF5505. KBU_SV1 was also positioned in proximity to *Enterobacteria* phage HK578 and *Sodalis* phage SO1. The congruence between TerL-based phylogeny and whole-proteome clustering supported the inferred evolutionary relationship of KBU_SV1 with related phages.

**Figure 11:**
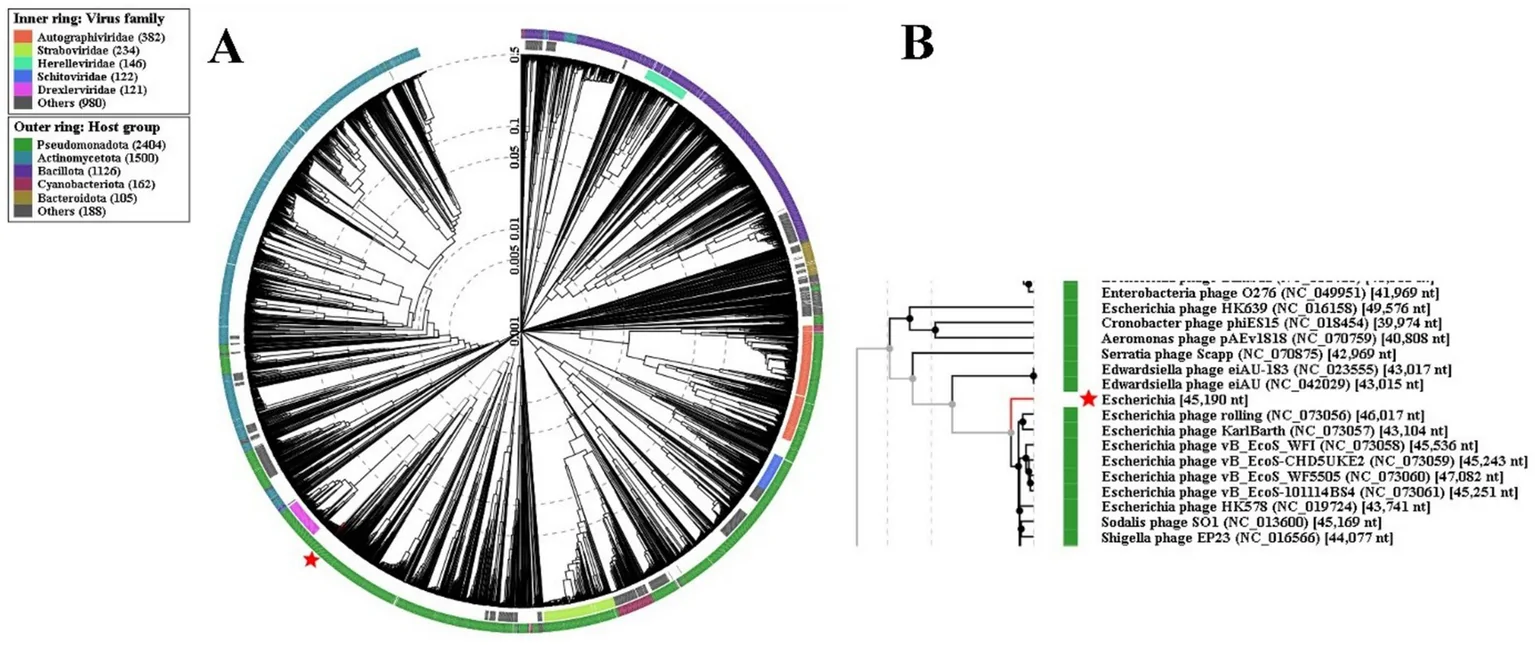
Proteomic tree of *Escherichia* phage KBU_SV1 and related bacteriophages generated using VipTree based on whole-genome similarities. **(A)** Circular proteomic tree showing the global distribution of viral genomes, with inner and outer rings representing virus families and host groups, respectively. **(B)** Enlarged view of the cluster containing KBU_SV1 (red star), highlighting its close relationship with *Escherichia* phages and related Enterobacterales-infecting viruses. The figure is constructed with VipTree version 4.1.

Independent genome-based taxonomic analysis using taxMyPhage assigned KBU_SV1 to the genus *Dhillonvirus* within the class *Caudoviricetes*. Family and subfamily ranks were not defined by taxMyPhage. Taken together, the phylogenetic and genome-based taxonomic analyses support the placement of KBU_SV1 within the genus *Dhillonvirus*. The complete genome sequence of *Escherichia phage* KBU_SV1 has been deposited in the GenBank database under accession number PZ050807.1.

## Discussion

The increasing prevalence of multidrug-resistant (MDR) *Escherichia coli* has intensified the search for alternative antibacterial strategies. Among these, bacteriophages have regained considerable attention because of their capacity to selectively target bacterial hosts, including antibiotic-resistant strains (Gordillo Altamirano and Barr, 2019; Kortright et al., 2019). In the present study, *Escherichia* phage KBU_SV1 was isolated from sewage and characterized with respect to its host spectrum, physicochemical stability, antibacterial and antibiofilm activities, genomic features, and *in vivo* efficacy. Collectively, the results identify KBU_SV1 as a newly isolated phage with a predicted virulent (lytic) lifestyle and several characteristics that support its further investigation as a potential antibacterial candidate against MDR *E. coli*.

Wastewater represents a rich reservoir of bacteriophages capable of infecting clinically relevant bacterial hosts (Cortes-Ortega et al., 2024). KBU_SV1 was successfully isolated from sewage using *E. coli* ATCC 25922 as the indicator and propagation host and produced well-defined plaques following successive plaque purification. Host-range screening demonstrated spot clearance against a subset of the tested *E. coli* isolates, including carbapenem-resistant clinical isolates, whereas clearance against *K. pneumoniae* was limited and no such effect was detected against the other bacterial species examined. Spot clearance alone does not establish productive phage infection, because clearing may also arise through mechanisms that do not produce progeny phages, such as lysis from without (Khan Mirzaei and Nilsson, 2015). The mechanism underlying the observed clearing was not determined in this study. Therefore, clearance on *K. pneumoniae* lawns should not be interpreted as confirmation of productive infection of this species. Plaque-formation assays and efficiency-of-plating measurements are needed to assess its susceptibility to productive infection by KBU_SV1. In general, strain-dependent differences in productive phage infection may reflect differences in surface receptors, including lipopolysaccharides, outer membrane proteins, and pili, as well as intracellular defense mechanisms such as restriction-modification and CRISPR-Cas systems (Dupuis et al., 2013; Hyman and Abedon, 2010; Oechslin, 2018). Although a restricted host spectrum may limit the applicability of a single phage as empirical therapy, phage host specificity can also enable selective targeting of susceptible bacterial populations while potentially limiting effects on non-target bacteria (Gordillo Altamirano and Barr, 2019; Hyman and Abedon, 2010).

KBU_SV1 retained infectivity over a relatively broad range of physicochemical conditions. Phage titers remained stable following incubation at 4, 23, and 37 °C but declined below detectable levels after exposure to 65 °C. Similarly, KBU_SV1 retained activity between pH 4 and 12 but lost detectable infectivity at pH 2. Temperature and pH are major determinants of phage stability because extreme conditions may alter capsid proteins, receptor-binding structures, or genomic integrity (Jończyk et al., 2011). The stability observed under moderate temperatures and across a broad pH interval therefore supports the robustness of KBU_SV1 under the experimental conditions tested, although formulation-specific stability and long-term storage were not evaluated in the present study.

Growth-reduction assays further demonstrated the antibacterial activity of KBU_SV1 against *E. coli* ATCC 25922. Although all tested MOIs maintained OD₅₉₅ values below 0.10 during the first 14 h, the subsequent growth response showed an apparent non-monotonic pattern. In particular, MOIs of 0.1 and 0.01 yielded lower OD₅₉₅ values at later time points than MOIs of 10 and 1, while MOI 100 also showed more sustained suppression than MOIs of 10 and 1. Regrowth during phage exposure is frequently observed in phage-bacterium systems and may be associated with the emergence or selection of phage-resistant subpopulations or other changes in phage-host interactions during prolonged exposure (Oechslin, 2018; Nacaroglu et al., 2026). However, these possibilities do not establish the cause of the specific MOI-dependent pattern observed here. Time-resolved viable bacterial counts and phage titers were not determined, and the phage susceptibility of regrown bacteria was not assessed. Consequently, the mechanism underlying this pattern remains unresolved, and the lower late OD₅₉₅ values at MOIs of 0.1 and 0.01 should not be interpreted as establishing the general superiority of lower MOIs or identifying an optimal MOI. Further experiments combining these measurements would be needed to distinguish the possible mechanisms.

KBU_SV1 also reduced established biofilm biomass and inhibited biofilm formation in both *E. coli* ATCC 25922 and the MDR clinical isolate Ec59, although the magnitude of the effect was greater against the reference strain. Differences in antibiofilm susceptibility between bacterial strains may reflect variation in extracellular polymeric substance composition, biofilm architecture, physiological heterogeneity, receptor accessibility, and phage-host interactions. These factors can substantially influence phage diffusion and infection within structured bacterial communities (Abedon, 2015). Importantly, the crystal violet assay used in the present study measures total surface-associated biofilm biomass rather than bacterial viability (Stepanović et al., 2007). These findings should therefore be interpreted as reductions in attached biofilm biomass rather than evidence of bacterial killing or complete biofilm eradication. Complementary approaches, including viable-cell enumeration, metabolic assays, or microscopic analysis, would be valuable for determining the extent to which KBU_SV1 reduces viable cells within established biofilms.

The *in vivo* findings provided additional evidence of biological activity under infection conditions. In the *Galleria mellonella* model, KBU_SV1 treatment significantly delayed mortality compared with the bacteria-only control, as demonstrated by Kaplan–Meier analysis. However, KBU_SV1 did not provide complete protection under the experimental conditions, and the eventual mortality observed in treated larvae indicates that its effect was predominantly reflected by delayed infection progression rather than sustained protection. The mechanism underlying the eventual mortality was not determined in the present study. Potential contributing factors may include insufficient phage persistence or amplification *in vivo*, suboptimal dosing, changes in bacterial susceptibility during infection, or incomplete control of the bacterial burden; however, these possibilities require direct experimental investigation. The absence of mortality in the phage-only group indicated no detectable toxicity of the administered phage preparation under the conditions tested. Nevertheless, *G. mellonella* is an invertebrate infection model and does not fully reproduce the physiological and immunological complexity of mammalian hosts (Tsai et al., 2016). Accordingly, these results should be considered preliminary *in vivo* evidence supporting further investigation rather than demonstration of therapeutic efficacy in mammalian hosts.

Genome analysis provided important additional information regarding the genomic characteristics and suitability of KBU_SV1 for further study. The phage possesses a 45,190-bp double-stranded DNA genome with a G + C content of 53.56%. Read mapping provided complete positional coverage of the assembly, while CheckV classified the genome as complete, with 100% estimated completeness, 0% contamination, and no detected host genes. In addition, a high-confidence 272-bp direct terminal repeat was identified at both genome termini. The identification of this DTR supports a defined terminal genome architecture; however, the present short-read sequencing data do not by themselves establish the DNA packaging mechanism, and no definitive packaging mechanism is therefore proposed. Genome-based lifestyle analyses further supported a predicted virulent (lytic) lifestyle. PhageAI classified KBU_SV1 as virulent, consistent with the PhageLeads assessment and the absence of canonical lysogeny-associated genes, including integrases, excisionases, and phage repressors. Moreover, complementary screening with ResFinder, VirulenceFinder, CARD/RGI, and VFDB detected no known antimicrobial resistance or virulence-associated determinants. In addition, an open reading frame initially annotated as a putative transposase was not supported by InterProScan domain analysis or BLASTp searches and was therefore considered a hypothetical protein, indicating that no mobile genetic element–associated gene was retained in the final annotation. These genomic characteristics are consistent with commonly considered criteria when evaluating phages for further therapeutic development, although the absence of known undesirable genes should not be interpreted as establishing clinical safety by itself (Fernández et al., 2019).

Comparative genomic analyses further clarified the taxonomic position of KBU_SV1. BLASTn analysis demonstrated high nucleotide similarity to several related *Escherichia* phages, including EPIMAM03 and Ioannina, while VIRIDIC showed greater than 95% intergenomic similarity with several closely related genomes. These results place KBU_SV1 within an existing species-level genomic cluster rather than supporting its designation as a novel bacteriophage species. This interpretation was consistent with the TerL-based phylogeny and whole-proteome comparison using VipTree. Independent analysis with taxMyPhage assigned KBU_SV1 to the genus *Dhillonvirus* within the class *Caudoviricetes*, whereas family and subfamily ranks were not defined. Together, these complementary genome-based approaches provide a more informative framework for taxonomic placement than morphology alone and are consistent with contemporary genome-based approaches to bacteriophage classification (Dion et al., 2020; Turner et al., 2021).

Several limitations of this study should be considered. First, host-range assessment was based on spot assays, and efficiency-of-plating measurements were not performed on the spot-test-positive clinical isolates. Because clearing in spot assays does not necessarily demonstrate productive infection, future studies should determine EOP values to more precisely define the productive host range of KBU_SV1. Second, a one-step growth experiment was not performed; consequently, adsorption kinetics, latent period, and burst size remain to be determined. Third, crystal violet staining quantifies total attached biofilm biomass but does not distinguish viable from non-viable cells; therefore, the observed reductions cannot be interpreted as evidence of bacterial killing or complete biofilm eradication. Fourth, bacterial regrowth during prolonged phage exposure was not investigated mechanistically, and the emergence of phage-resistant variants should be examined in future studies. Fifth, although genomic analyses strongly supported assembly completeness and identified a 272-bp DTR, the DNA packaging mechanism was not experimentally resolved. Sixth, the growth-reduction and *in vivo* experiments were performed with the reference strain *E. coli* ATCC 25922, which provides standardized conditions and allows the findings to be independently reproduced, whereas the activity of KBU_SV1 against carbapenem-resistant *E. coli* was demonstrated in the host-range screening and in the biofilm assays with the clinical isolate Ec59; these experiments should therefore be extended to a broader panel of carbapenem-resistant clinical isolates. Finally, regarding the *in vivo* assessment, although the phage preparation was washed via high-speed centrifugation and pelleting to reduce soluble bacterial contaminants, the exact endotoxin levels of the final preparation were not quantified. Furthermore, the *G. mellonella* experiment represents an initial non-mammalian *in vivo* assessment and showed delayed mortality rather than complete protection. Additional studies incorporating optimized dosing, phage pharmacodynamics, bacterial and phage enumeration during infection, broader panels of clinical isolates, and appropriate mammalian infection models such as murine models will therefore be required before the therapeutic applicability of KBU_SV1 can be established.

## Conclusion

This study characterized *Escherichia* phage KBU_SV1 as a newly isolated phage with antibacterial and antibiofilm activity against *E. coli*, including carbapenem-resistant clinical isolates. KBU_SV1 remained infective across a relatively broad range of pH conditions and at moderate temperatures, reduced established biofilm biomass, inhibited biofilm formation, and significantly delayed mortality in the *Galleria mellonella* infection model. Genomic analyses supported a predicted virulent (lytic) lifestyle and revealed no known antimicrobial resistance or virulence-associated determinants. Comparative genomic analyses assigned KBU_SV1 to the genus *Dhillonvirus* and placed it within an existing species-level genomic cluster. Collectively, these findings support the further investigation of KBU_SV1 as a potential phage-based antibacterial candidate against multidrug-resistant *E. coli*. However, additional studies addressing productive host range, phage replication parameters, resistance development, optimized dosing, and efficacy in mammalian infection models are required to determine its therapeutic applicability.

## Data Availability

The data analyzed in this study are available in the NCBI Genbank (https://www.ncbi.nlm.nih.gov/genbank) under accession number PZ050807.
