# Supplementary material for: Biological and genomic characterization of Escherichia phage KBU_SV1 active against carbapenem-resistant Escherichia coli
Source: Front Microbiol. 2026 Oct 15;17:1927801. doi: 10.3389/fmicb.2026.1927801 (PMC13717695; doi:10.3389/fmicb.2026.1927801)
Supplement: Supplementary file 1 [file Data_Sheet_1.docx]

**SUPPLEMENTARY DATA**

**Biological and Genomic Characterization of *Escherichia* Phage KBU_SV1 Active Against Carbapenem-Resistant *Escherichia coli***

Sevil Öztaş¹*, Abdulkerim Karaynir², Rümeysa Gülsu Özkan ^5,6^, Bülent Bozdoğan²^,^³, Devrim

Dündar⁴

**Supplementary Figure S1.** Restriction profile of KBU_SV1 genomic DNA (Left) Intact phage genomic DNA. (Right) Band profile of the phage DNA after double digestion with *EcoRI* and *HindIII* restriction enzymes. M: Lambda-PstI molecular weight marker

**Supplementary Table S1.** Host range of bacteriophage KBU_SV1 and phenotypic and available genotypic characteristics of isolates

| ***Isolates*** | ***Bacteria type and  resistance genes*** | **Sample type** | **CRO** | **CIP** | **SXT** | **IPM** | **MEM** | **ETP** | **GEN** | **COL** | **TZP** | **PLZ** | **CZA** | **KBU_SV1 phage** |
| --- | --- | --- | --- | --- | --- | --- | --- | --- | --- | --- | --- | --- | --- | --- |
| E2 | *E. coli (bla_NDM_, bla_CTX_-_M1_, bla_TEM_, bla_OXA1-LIKE_)* | Wound specimen | R | R | R | S | R | R | R | S | R | R | R | - |
| E3A | *E. coli (bla_VIM_, bla_OXA1-LIKE_)* | Urine | R | R | R | S | S | R | R | S | R | S | R | - |
| E4 | *E. coli (bla_NDM_, bla_CTX-M1_, bla_TEM_, bla_OXA1-LIKE_)* | Urine | R | S | S | S | S | R | S | R | R | R | R | **++** |
| E6 | *E. coli (bla_OXA-48_, bla_CTXM-9_)* | Urine | R | R | R | R | R | R | R | R | R | I | S | **++** |
| E8 | *E. coli (bla_OXA-48,_ bla_CTXM-1_, bla_TEM_, bla_SHV_, bla_OXA1-LIKE_)* | Wound specimen | R | R | S | R | R | R | R | S | R | R | S | - |
| E9 | *E. coli (bla_VIM_, bla_OXA1-LIKE_)* | Aspirate | R | R | R | R | S | R | R | S | R | S | R | - |
| E10 | *E. coli (bla_OXA-48_, bla_CTXM-1_, bla_TEM_, bla_SHV_, bla_OXA1-LIKE_)* | Wound specimen | R | S | R | R | R | R | S | S | S | R | S | - |
| E11 | *E. coli (bla_OXA-48_, bla_CTXM-9_)* | Urine | R | R | R | R | S | R | S | R | R | 8 | S | **+++** |
| E13 | *E. coli (bla_NDM_, bla_OXA-48_, bla_CTX-M1_, bla_TEM_, bla_OXA1-LIKE_)* | Urine | S | R | S | R | R | R | S | S | R | R | R | - |
| E14 | *E. coli (bla_OXA-48_, bla_IMP_, bla_KPC_, bla_CTXM-1_, bla_OXA1-LIKE_)* | Blood | R | R | R | S | S | R | R | R | R | I | R | - |
| E15 | *E. coli (bla_NDM_, bla_CTX-M1_, bla_SHV_)* | Wound specimen | R | R | R | R | R | R | R | S | S | R | R | **++** |
| E16 | *E. coli (bla_NDM_, bla_OXA-48_, bla_CTX-M1_, bla_TEM_, bla_SHV_, bla_OXA1-LIKE_)* | Wound specimen | R | R | R | R | R | R | S | S | R | R | R | - |
| E17 | *E. coli (bla_NDM_, bla_OXA-48_, bla_CTX-M1_, bla_TEM_, bla_SHV_, bla_OXA1-LIKE_)* | Urine | R | S | R | R | R | R | S | R | R | R | R | - |
| E19 | *E. coli (bla_NDM_, bla_OXA-48_, bla_CTX-M1_, bla_TEM_, bla_OXA1-LIKE_)* | Urine | R | R | R | R | R | R | R | R | R | R | R | - |
| E20 | *E. coli (bla_OXA-48_, bla_IMP_, bla_CTXM-1_)* | Wound specimen | R | R | R | S | R | S | S | R | R | 8 | S | - |
| E21 | *E. coli (_blaOXA-48_)* | Urine | R | R | R | R | R | R | R | S | R | R | R | - |
| E22 | *E. coli (bla_NDM_, bla_KPC_, bla_VIM_)* | Urine | S | S | R | R | R | R | S | S | R | 0.25 | R | **+** |
| E26 | *E. coli (bla_OXA-48_, bla_TEM_, bla_SHV_)* | Wound specimen | R | R | S | R | R | R | R | S | R | R | S | - |
| E59 | *E. coli (bla_NDM_, bla_CTXM-1_, bla_SHV_)* | Urine | R | R | R | R | R | R | R | R | R | R | R | + |
| T23 | *E. coli (bla_OXA-48_, bla_CTXM-1_, bla_TEM_, bla_SHV_, bla_OXA1-LIKE_)* | Wound specimen | R | R | R | R | R | R | S | R | R | 0.5 | S | + |
| K2 | *K.pneumonia (bla_OXA-48_, bla_CTXM-1_, bla_TEM_, bla_SHV_, bla_OXA1-LIKE_)* | Urine | R | R | R | S | R | R | S | S | R | R | S | - |
| K3 | *K.pneumonia (bla_OXA-48_, bla_CTXM-1_, bla_TEM_, bla_SHV_, bla_OXA1-LIKE_)* | Urine | R | S | S | R | R | R | S | R | R | I | S | - |
| K4 | *K.pneumonia (bla_OXA-48_, bla_CTXM-1_, bla_TEM_, bla_SHV_, bla_OXA1-LIKE_)* | Urine | R | R | R | S | R | R | S | R | R | R | S | - |
| K5 | *K.pneumonia (bla_OXA-48_, bla_CTXM-1_, bla_TEM_, bla_SHV_, bla_OXA1-LIKE_)* | Wound specimen | S | S | S | S | I | R | S | S | S | R | S | - |
| K7 | *K.pneumonia (bla_OXA-48_, bla_CTXM-1_, bla_SHV_, bla_OXA1-LIKE_)* | Pus | R | R | R | R | R | R | R | R | R | I | S | - |
| K8 | *K.pneumonia (bla_OXA-48_, bla_CTXM-1_, bla_TEM_, bla_SHV_, bla_OXA1-LIKE_)* | Pleural fluid | R | R | R | R | R | R | S | S | R | R | S | - |
| K11 | *K.pneumonia (bla_OXA-48_, bla_CTXM-1_, bla_TEM_, bla_SHV_, bla_OXA1-LIKE_)* | Urine | R | R | S | R | R | R | S | R | R | R | S | - |
| K13 | *K.pneumonia (bla_OXA-48_, bla_CTXM-1_, bla_TEM_, bla_SHV_, bla_OXA1-LIKE_)* | BAL | R | R | R | R | R | R | S | S | R | R | S | - |
| K14 | *K.pneumonia (bla_OXA-48_, bla_CTXM-1_, bla_TEM_, bla_SHV_, bla_OXA1-LIKE_)* | Urine | R | R | R | R | R | R | R | R | R | S | S | - |
| K15 | *K.pneumonia (bla_OXA-48_, bla_CTXM-1_, bla_TEM_, bla_SHV_, bla_OXA1-LIKE_)* | Urine | R | I | R | R | R | R | S | S | R | S | S | - |
| K21 | *K.pneumonia (bla_IMP_, bla_KPC_, bla_CTXM-1_, bla_TEM_, bla_SHV_, bla_OXA1-LIKE_)* | Urine | R | R | R | S | S | R | S | R | R | S | S | - |
| K22 | *K.pneumonia (bla_NDM_, bla_CTXM-1_, bla_TEM_, bla_SHV_, bla_OXA1-LIKE_)* | Wound specimen | R | R | R | R | R | R | R | R | R | R | R | - |
| K23 | *K.pneumonia (bla_OXA-48_, bla_NDM_, bla_CTXM-1_, bla_TEM_, bla_SHV_, bla_OXA1-LIKE_)* | Urine | S | R | R | R | R | R | R | R | R | R | R | - |
| K24 | *K.pneumonia (bla_NDM_, bla_SHV_)* | Tracheal aspirate | R | S | S | R | R | R | R | S | R | R | R | - |
| K26 | *K.pneumonia (bla_OXA-48_, bla_CTXM-1_, bla_TEM_, bla_SHV_, bla_OXA1-LIKE_)* | Urine | R | R | R | R | R | R | S | S | R | I | S | - |
| K27 | *K.pneumonia (bla_NDM_, bla_CTXM-1_, bla_SHV_, bla_OXA1-LIKE_)* | Urine | R | R | R | R | R | R | R | S | R | R | R | - |
| K29 | *K.pneumonia (bla_OXA-48_, bla_TEM_, bla_SHV_, bla_OXA1-LIKE_)* | Urine | R | R | R | R | R | R | R | S | R | R | S | - |
| K30 | *K.pneumonia (bla_OXA-48_, bla_TEM_, bla_SHV_)* | CVP catheter | S | R | S | R | S | R | S | S | R | S | S | - |
| K33 | *K.pneumonia (bla_OXA-48_, bla_CTXM-1_, bla_SHV_)* | Urine | S | S | S | R | R | R | S | S | R | S | R | **+** |
| C3 | *K.pneumonia (bla_NDM_, bla_CTXM-1_, bla_SHV_, bla_OXA1-LIKE_)* | Urine | R | R | S | R | R | R | R | R | R | R | R | **+** |
| **Other strains** | | | | | | | | | | | | | | |
| ***Isolates*** | | ***Bacteria type*** | | ***Infectivity*** | | | ***Isolates*** | | ***Bacteria type*** | | | ***Infectivity*** | | |
| E1 | | *(CSEC) E. coli* | | - | | | KpK1 | | *(CSKP) K.pneumonia* | | | - | | |
| E2 | | *(CSEC) E. coli* | | - | | | KpK2 | | *(CSKP) K.pneumonia* | | | - | | |
| E3 | | *(CSEC) E. coli* | | - | | | KpK3 | | *(CSKP) K.pneumonia* | | | - | | |
| E4 | | *(CSEC) E. coli* | | - | | | KpK4 | | *(CSKP) K.pneumonia* | | | - | | |
| E5 | | *(CSEC) E. coli* | | - | | | KpK5 | | *(CSKP) K.pneumonia* | | | - | | |
| E6 | | *(CSEC) E. coli* | | - | | | KpK6 | | *(CSKP) K.pneumonia* | | | - | | |
| E7 | | *(CSEC) E. coli* | | - | | | KpK7 | | *(CSKP) K.pneumonia* | | | - | | |
| E8 | | *(CSEC) E. coli* | | + | | | KpK8 | | *(CSKP) K.pneumonia* | | | - | | |
| E9 | | *(CSEC) E. coli* | | + | | | KpK9 | | *(CSKP) K.pneumonia* | | | - | | |
| E10 | | *(CSEC) E. coli* | | - | | | KpK10 | | *(CSKP) K.pneumonia* | | | - | | |
| E11 | | *(CSEC) E. coli* | | - | | | KpK11 | | *(CSKP) K.pneumonia* | | | - | | |
| E12 | | *(CSEC) E. coli* | | - | | | KpK12 | | *(CSKP) K.pneumonia* | | | - | | |
| E13 | | *(CSEC) E. coli* | | - | | | KpK13 | | *(CSKP) K.pneumonia* | | | - | | |
| E14 | | *(CSEC) E. coli* | | - | | | KpK14 | | *(CSKP) K.pneumonia* | | | - | | |
| E15 | | *(CSEC) E. coli* | | - | | | KpK15 | | *(CSKP) K.pneumonia* | | | - | | |
| E16 | | *(CSEC) E. coli* | | - | | | KpK16 | | *(CSKP) K.pneumonia* | | | - | | |
| E17 | | *(CSEC) E. coli* | | - | | | KpK17 | | *(CSKP) K.pneumonia* | | | **+** | | |
| E18 | | *(CSEC) E. coli* | | + | | | KpK18 | | *(CSKP) K.pneumonia* | | | - | | |
| E19 | | *(CSEC) E. coli* | | + | | | KpK19 | | *(CSKP) K.pneumonia* | | | + | | |
| E20 | | *(CSEC) E. coli* | | - | | | KpK20 | | *(CSKP) K.pneumonia* | | | - | | |
| ATCC 25922 | | *(CSEC) E. coli* | | + | | |  | |  | | |  | | |
| EF1 | | *E. faecalis* | | - | | | EF11 | | *E. faecalis* | | | - | | |
| EF2 | | *E. faecalis* | | - | | | EF12 | | *E. faecalis* | | | - | | |
| EF3 | | *E. faecalis* | | - | | | EF13 | | *E. faecalis* | | | - | | |
| EF4 | | *E. faecalis* | | - | | | EF14 | | *E. faecalis* | | | - | | |
| EF5 | | *E. faecalis* | | - | | | EF15 | | *E. faecalis* | | | - | | |
| EF6 | | *E. faecalis* | | - | | | EF16 | | *E. faecalis* | | | - | | |
| EF7 | | *E. faecalis* | | - | | | EF17 | | *E. faecalis* | | | - | | |
| EF8 | | *E. faecalis* | | - | | | EF18 | | *E. faecalis* | | | - | | |
| EF9 | | *E. faecalis* | | - | | | EF19 | | *E. faecalis* | | | - | | |
| EF10 | | *E. faecalis* | | - | | | EF20 | | *E. faecalis* | | | - | | |
| ATCC 19606 | | *A. baumannii* | | - | | | ATCC 27853 | | *P. aeruginosa* | | | - | | |
| ATCC 29213 | | *S. aureus* | | - | | | ATCC 29212 | | *E. faecalis* | | | - | | |
| ATCC 700603 | | *K. pneumoniae* | | - | | | ATCC 35984 | | *S. epidermitis* | | | - | | |

**Abbreviations:** CRO, ceftriaxone; CAZ, ceftazidime; CZA, ceftazidime-avibactam; CIP, ciprofloxacin; SXT, trimethoprim-sulfamethoxazole; IPM, imipenem; MEM, meropenem; ETP, ertapenem; GEN, gentamicin; COL, colistin; TZP, piperacillin-tazobactam; PLZ, plazomicin. **S**, susceptible; **I**, susceptible, increased exposure; **R**, resistant.
